# Latent TGFβ-binding proteins 1 and 3 protect the larval zebrafish outflow tract from aneurysmal dilatation

**DOI:** 10.1242/dmm.046979

**Published:** 2022-03-28

**Authors:** Maryline Abrial, Sandeep Basu, Mengmeng Huang, Vincent Butty, Asya Schwertner, Spencer Jeffrey, Daniel Jordan, Caroline E. Burns, C. Geoffrey Burns

**Affiliations:** 1 Cardiovascular Research Center, Department of Cardiology, Massachusetts General Hospital, Charlestown, MA 02129, USA; 2 Harvard Medical School, Boston, MA 02115, USA; 3 Division of Basic and Translational Cardiovascular Research, Department of Cardiology, Boston Children's Hospital, Boston, MA 02115, USA; 4 BioMicroCenter, Department of Biology, Massachusetts Institute of Technology, Cambridge, MA 02139, USA; 5 Harvard Stem Cell Institute, Cambridge, MA 02138, USA

**Keywords:** Zebrafish, Outflow tract, Thoracic aortic aneurysm, LTBP proteins, TGFβ signaling

## Abstract

Aortic root aneurysm is a common cause of morbidity and mortality in Loeys-Dietz and Marfan syndromes, where perturbations in transforming growth factor beta (TGFβ) signaling play a causal or contributory role, respectively. Despite the advantages of cross-species disease modeling, animal models of aortic root aneurysm are largely restricted to genetically engineered mice. Here, we report that zebrafish devoid of the genes encoding latent-transforming growth factor beta-binding protein 1 and 3 (*ltbp1* and *ltbp3*, respectively) develop rapid and severe aneurysm of the outflow tract (OFT), the aortic root equivalent. Similar to syndromic aneurysm tissue, the distended OFTs display evidence for paradoxical hyperactivated TGFβ signaling. RNA-sequencing revealed significant overlap between the molecular signatures of disease tissue from mutant zebrafish and a mouse model of Marfan syndrome. Moreover, chemical inhibition of TGFβ signaling in wild-type animals phenocopied mutants but chemical activation did not, demonstrating that TGFβ signaling is protective against aneurysm. Human relevance is supported by recent studies implicating genetic lesions in *LTBP3* and, potentially, *LTBP1* as heritable causes of aortic root aneurysm. Ultimately, our data demonstrate that zebrafish can now be leveraged to interrogate thoracic aneurysmal disease and identify novel lead compounds through small-molecule suppressor screens.

This article has an associated First Person interview with the first author of the paper.

## Abstract (precis)

**Summary:** This study describes one of the first zebrafish models of thoracic aortic aneurysm, arising from mutations in *ltbp1* and *ltbp3*, genes that regulate TGFβ signaling.

## INTRODUCTION

Thoracic aortic aneurysms (TAAs) are focal and progressive expansions of the aortic wall that occur most commonly in the aortic root or ascending aorta (Hiratzka et al., 2010). Risk factors include hypertension, bicuspid aortic valve and *de novo* or inherited mutations responsible for non-syndromic or syndromic forms of the disease. Syndromic TAAs occur in the context of Loeys-Dietz syndrome (LDS) or Marfan syndrome (MFS), which arise from mutations in genes encoding transforming growth factor beta (TGFB, hereafter referred to as TGFβ)*-*signaling components or the extracellular matrix (ECM) protein fibrillin-1 (FBN1), respectively. TAAs become problematic when a tear in the innermost layer creates a false lumen in the aortic wall. Termed an aortic dissection, this clinical event is a medical emergency due to the risk of impaired blood flow and aortic rupture. When individuals are diagnosed with subclinical TAAs, routine monitoring begins, and attempts are made to slow aneurysm progression with β blockers (anti-hypertensives) or angiotensin II type I receptor blockers (anti-hypertensives with indirect TGFβ inhibition). However, in many cases, aneurysm growth continues and surgical replacement with a synthetic graft becomes necessary to prevent dissection.

Latent-transforming growth factor beta-binding proteins (LTBPs) regulate the bioavailability of TGFβ ligands – i.e. TGFβ family members TGFB1-3 – by anchoring small latent complexes (SLCs), composed of a mature ligand and latency-associated peptides, to the ECM until the ligand becomes activated and released through one of several mechanisms (Rifkin et al., 2018). Because LTBPs are required for both the secretion and activation of TGFβ ligands, knocking out any given LTBP gene is predicted to reduce or eliminate any downstream TGFβ signaling events normally facilitated by the respective LTBP protein. Once activated, TGFβ ligands induce TGFβ signaling in surrounding cells through a series of well-described molecular events (Derynck and Budi, 2019). These include phosphorylation of TGFβ receptor type-1 (TGFBR1) by TGFβ receptor type-2 (TGFBR2) and subsequent phosphorylation of SMAD family members 2 and 3 (SMAD2 and SMAD3, respectively) by TGFBR1. Phosphorylated SMAD2/3 proteins (pSMAD2/3) associate with the co-SMAD SMAD4 and translocate together into the nucleus where they modulate transcription of TGFβ target genes.

A large body of work has identified alterations in TGFβ signaling as both an instigator of TAAs and feature of disease tissue in MFS and LDS (reviewed by Lindsay and Dietz, 2014; Pinard et al., 2019; Verstraeten et al., 2017). However, apparent inconsistencies between the observed status of TGFβ signaling in aneurysm tissue and the known consequences of LDS and MFS mutations on signaling activity have remained a source of confusion and controversy in the field (Cook et al., 2015; Mallat et al., 2017; Wei et al., 2017). Specifically, aneurysm tissue from MFS and LDS patients and genetically engineered mouse models exhibit the hallmarks of hyperactivated TGFβ signaling (Gomez et al., 2009; Habashi et al., 2006; Holm et al., 2011; Lindsay et al., 2012; Loeys et al., 2005). However, LDS mutations, which occur in genes encoding one of several components of the TGFβ signaling pathway – i.e. *TGFB2* (Lindsay et al., 2012), *TGFBR1* (Loeys et al., 2005), *TGFBR2* (Loeys et al., 2005) and *SMAD3* (Laar et al., 2011) – are loss-of-function mutations that undermine TGFβ signaling (MacFarlane et al., 2019; Pinard et al., 2019).

The mutated gene in MFS, *FBN1*, encodes a protein that interacts with LTBP proteins and anchors large latent complexes (LLCs), composed of an LTBP protein and SLC, to the ECM until the ligand becomes activated by release into the surrounding tissue (Isogai et al., 2003; Zilberberg et al., 2012). Therefore, it was hypothesized that mutated FBN1 proteins in MFS fail to tether LLCs to the ECM (Neptune et al., 2003), ultimately leading to unrestrained TGFβ signaling as the driver of aneurysm (Habashi et al., 2006). While this hypothesis accounted for the hallmarks of hyperactivated TGFβ signaling in aneurysm tissue, an alternative view posits instead, that *FBN1* mutations undermine TGFβ signaling because ECM tethering is a prerequisite for ligand activation (Rifkin et al., 2018). Therefore, it remains plausible that aneurysm susceptibility in MFS stems from reduced TGFβ signaling, as is the case for LDS (Cook et al., 2015; Mallat et al., 2017; Rifkin et al., 2018). The paradoxical hallmarks of hyperactivated TGFβ signaling have been attributed to reactive compensatory mechanisms (Cook et al., 2015; Holm et al., 2011; Lindsay and Dietz, 2011; MacFarlane et al., 2019) or non-specific secondary responses to disease progression (Mallat et al., 2017; Milewicz et al., 2017). Nonetheless, the paradox remains largely unresolved from a molecular perspective. Ultimately, given the uncertainties surrounding molecular pathogenesis and lack of curative medical treatments, additional fundamental insights and new lead compounds are required to improve the therapeutic options for TAAs.

For studying aortic root pathologies, zebrafish is an underutilized but relevant model organism, given that many parallels can be drawn between the aortic root in humans and the comparable structure in zebrafish, termed the outflow tract (OFT). These structures are analogous and homologous on the basis of similarities in anatomic location, tissue architecture and embryonic origins. In humans, the aortic root forms the conduit between the left ventricle and the ascending aorta (Hiratzka et al., 2010). In zebrafish, the OFT connects the single ventricle to the ventral aorta and pharyngeal arch arteries (Anderson et al., 2008; Grimes et al., 2006; Guner-Ataman et al., 2013; Paffett-Lugassy et al., 2017). In higher vertebrates, the aortic root derives from a transient structure termed the embryonic OFT, which resembles the zebrafish OFT because it connects the pre-septation primitive ventricle to the aortic sac and pharyngeal arch arteries (Webb et al., 2003).

OFTs in all vertebrates, including those that become remodeled, exhibit a conserved pattern of muscle composition characterized by a proximal myocardial compartment that partially overlaps with a distal smooth muscle segment (Grimes et al., 2010). In zebrafish, the myocardial collar is relatively short compared to the adjacent segment of elastin2 (Elnb, hereafter referred to as Eln2)-positive smooth muscle known as the bulbus arteriosus (Grimes et al., 2006, 2010; Hami et al., 2011; Miao et al., 2007; Paffett-Lugassy et al., 2017). In all species examined, the OFT myocardium derives from *NKX2.5*-positive second heart field (SHF) progenitors within the cores of anterior pharyngeal arches (Guner-Ataman et al., 2013; Kelly et al., 2001; Lescroart et al., 2010; Mjaatvedt et al., 2001; Paffett-Lugassy et al., 2017; Stanley et al., 2002; Tirosh-Finkel et al., 2006; Waldo et al., 2001). The distal smooth muscle segment derives from two sources that make characteristic spatial contributions. The proximal smooth muscle derives from *NKX2.5*-positive SHF progenitors, whereas distal smooth muscle originates from cardiac neural crest progenitors (Cavanaugh et al., 2015; Guner-Ataman et al., 2013; Harmon and Nakano, 2013; Paffett-Lugassy et al., 2013; Verzi et al., 2005; Waldo et al., 2005).

After OFT morphogenesis, the simple anatomy of the zebrafish OFT does not change appreciably during growth (Grimes et al., 2006; Hu et al., 2001). However, in higher vertebrates, the embryonic OFT undergoes extensive remodeling, which includes septation to accommodate the parallel pulmonary and systemic circulations (Kirby, 2007). During remodeling, the OFT myocardium largely regresses but the residual muscle becomes the sub-pulmonary and sub-aortic myocardium (Bajolle et al., 2006, 2008; Waldo et al., 2005). The SHF-derived OFT smooth muscle comes to inhabit the middle layer (i.e. the tunica media) of the pulmonary artery and aorta at their roots (Harmon and Nakano, 2013; Sawada et al., 2017; Verzi et al., 2005; Waldo et al., 2005). Therefore, the OFT myocardium in zebrafish is homologous and analogous to the subaortic and subpulmonary myocardium in higher vertebrates. Moreover, the OFT smooth muscle, largely derived from the SHF (Cavanaugh et al., 2015; Guner-Ataman et al., 2013; Paffett-Lugassy et al., 2017), is akin to the smooth muscle at the bases of the pulmonary artery and aorta (Grimes et al., 2006; Waldo et al., 2005). Given these similarities, the zebrafish OFT is an appropriate tissue for modeling diseases that affect the roots of the pulmonary artery and aorta, the latter of which is highly susceptible to aneurysm in the human population.

Few studies have utilized zebrafish to model aortic root pathologies, which is unfortunate given that zebrafish is a powerful model organism for developmental genetics, disease modeling and lead compound discovery through small-molecule suppressor screens. Three previous studies have inactivated TAA susceptibility genes in zebrafish (Doyle et al., 2012; Gould et al., 2019; Guo et al., 2015) and, although defects in cardiovascular development or hemodynamics were reported, no study documented expansion of the OFT. Here we report that knocking out the TGFβ regulators *ltbp1* and *ltbp3* causes rapid aneurysmal dilatation of the OFT, which exhibits several hallmarks of TAAs in humans.

## RESULTS

### *ltbp1* and *ltbp3* function redundantly in zebrafish to protect the OFT from aneurysmal dilatation, and the ventricle from chamber dilation

We reported previously that morpholino-mediated knockdown of *ltbp3* in zebrafish embryos undermines the contribution of SHF progenitors to ventricular myocardium and OFT smooth muscle (Zhou et al., 2011). To characterize this phenotype further without relying on morpholinos, we employed transcription activator-like (TAL) effector nucleases (TALENs) to induce mutations in exon 3 of the *ltbp3* locus. We isolated a putative null allele, *ltbp3^fb28^*, which carries a 7 bp deletion (see Materials and Methods) and is predicted to encode a severely truncated protein incapable of associating with TGFβ ligands (Fig. S1A). Surprisingly, animals homozygous for *ltbp3^fb28^* are grossly indistinguishable from their siblings during embryonic and larval stages. Nonetheless, because SHF phenotypes can be subtle (Jahangiri et al., 2016), we analyzed mutant hearts at 48 h post fertilization (hpf), when SHF defects become evident as a reduction in the number of ventricular cardiomyocytes (Zhou et al., 2011). Quantification of ventricular cardiomyocytes revealed equivalent numbers in mutants and siblings (Fig. S1B-D). Atrial cardiomyocyte numbers were also unaffected in mutant hearts (Fig. S1B-D). To investigate maternal effects, we generated maternal-zygotic mutants and did not observe any gross abnormalities during embryonic or larval stages. Taken together, these data demonstrate that *ltbp3* null animals are devoid of the SHF defects we documented previously in *ltbp3* morphants.

One potential explanation is that *ltbp3fb^28^* is not a bona fide null allele. However, we find this to be unlikely for several reasons. First, the mutant protein is predicted to contain only 13% of the wild-type amino acid sequence and be incapable of associating with TGFβ ligands (Fig. S1A). Second, quantitative PCR (qPCR) uncovered an 80% reduction of *ltbp3* levels in homozygous animals (Fig. S1E), which is consistent with nonsense mediated decay (NMD). Lastly, despite the absence of gross phenotypes during embryonic and larval stages, *ltbp3* null zebrafish subsequently developed spinal curvatures (Fig. S1F), a phenotype shared with *LTBP3* null mice (Dabovic et al., 2002). Taken together, these properties suggest that *ltbp3fb^28^* is a null allele.

A second potential explanation is genetic compensation or transcriptional adaptation, whereby null animals are uniquely capable of upregulating one or several closely related genes to compensate for the deleted gene (El-Brolosy et al., 2019; Ma et al., 2019). Among the four LTBP proteins in higher vertebrates, LTBP3 shares the most functional similarity to LTBP1 in that both proteins bind and regulate the bioavailability of all three TGFβ ligands (Rifkin et al., 2018). LTBP4 only binds TGFB1 and LTBP2 does not bind ligands. Therefore, we evaluated the relative expression levels of *ltbp1* between *ltbp3* null animals and control siblings and documented a 43% reduction in the mutant (Fig. S1G). These data rule out transcriptional adaptation of *ltbp1* as a compensatory mechanism in *ltbp3* null zebrafish.

**Fig. 1.**
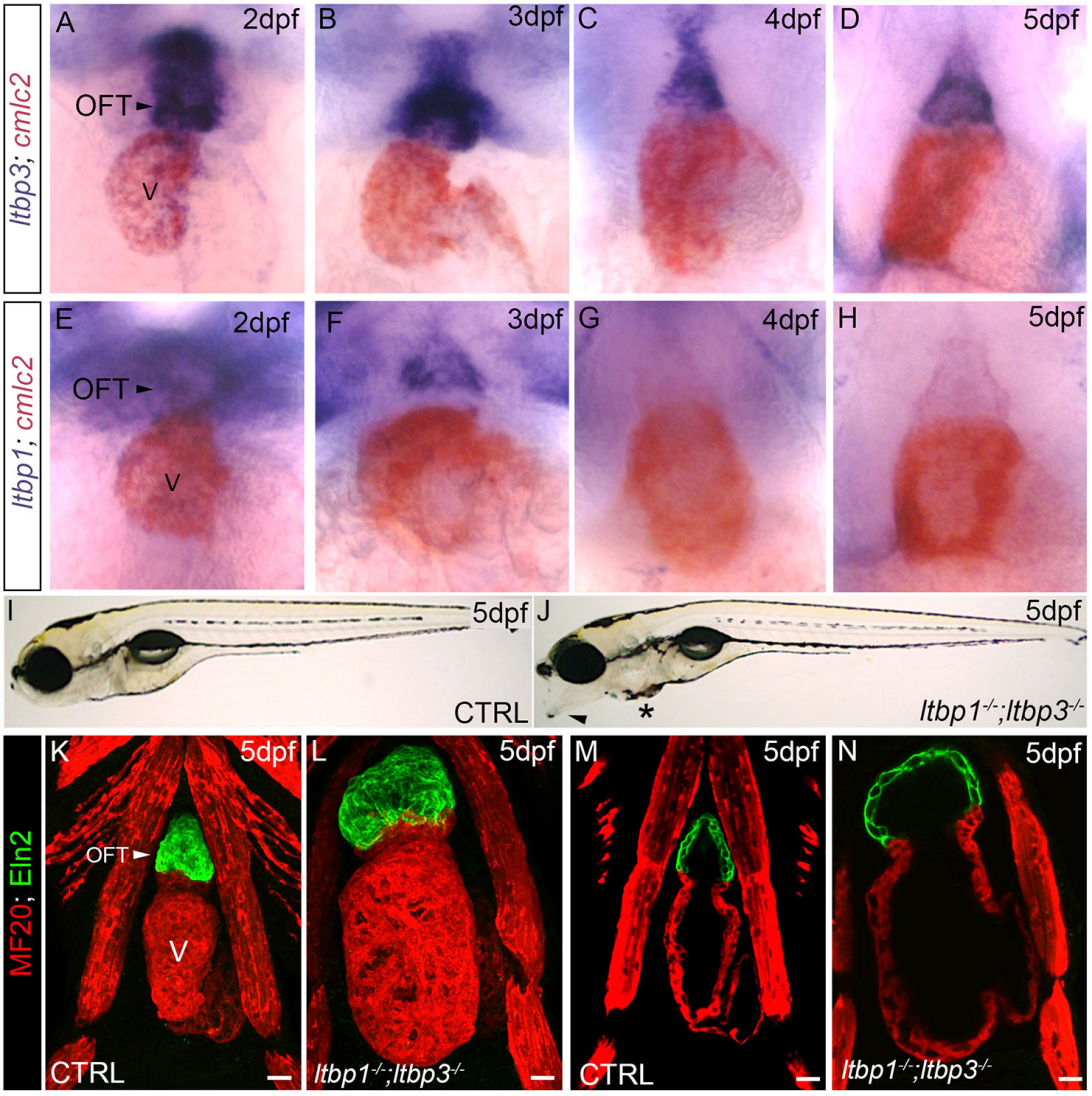
***ltbp1*, *ltbp3* DKO zebrafish exhibit OFT aneurysm and ventricular dilation.** (A-H) Brightfield images of hearts in wild-type zebrafish at 2 dpf (A,E), 3 dpf (B,F), 4 dpf (C,G), and 5 dpf (D,H) processed for double whole-mount *in situ* hybridization to detect *ltbp3* (A-D, blue signal) or *ltbp1* (E-H, blue signal) transcripts and the myocardial transcript *cmlc2* (*myl7*; A-H; red signal). Little to no variation was observed between animals within each group (*n*>10/group). (I,J) Brightfield images of 5 dpf control (CTRL; I) and *ltbp1^−/−^; ltbp3^−/−^* (J) larvae. Arrowhead and * in J highlight jaw protrusion and mild pericardial edema, respectively, observed in double mutants. (K-N) Confocal images of hearts in 5 dpf CTRL (K,M) and *ltbp1^−/−^; ltbp3^−/−^* (L,N) larvae double immunostained for striated muscle (MF20, red) and Eln2-positive OFT smooth muscle (green). The single optical sections shown in M and N were taken from the images shown in K and L. V, ventricle. Scale bars: 20 µm.

However, these data also highlighted an unappreciated dependency of robust *ltbp1* expression on *ltbp3* function, which brought up the possibility that *ltbp1* and *ltbp3* expression might overlap spatiotemporally. To evaluate this possibility in the heart, we performed *in situ* hybridization for both transcripts daily between 2 days post fertilization (dpf) and 5 dpf and compared their distributions to the myocardial marker *cmlc2/myl7* (Yelon et al., 1999). At all stages analyzed, *ltbp3* transcripts were observed anterior to the ventricle in the OFT (Fig. 1A-D). *ltbp1* transcripts were detected overlapping the same region on 2 dpf and 3 dpf but not thereafter (Fig. 1E-H). Little to no expression of either transcript was observed in the ventricle (Fig. 1A-H). These overlapping expression patterns brought up the possibility that *ltbp3* and *ltbp1* perform redundant functions in OFT development, maturation or homeostasis.

To test this hypothesis, we created and characterized *ltbp1, ltbp3* double knockout (DKO; *ltbp1^−/−^; ltbp3^−/−^*) animals. We employed CRISPR/Cas9-mediated genome editing to induce mutations in exon 14 of the *ltbp1* locus. We isolated a novel allele, *ltbp1^fb29^*, which contains an 8 bp deletion (see Materials and Methods) and is predicted to encode a significantly truncated protein incapable of associating with TGFβ ligands (Fig. S2A). qPCR revealed an 86% reduction of *ltbp1* levels in homozygous animals (Fig. S2B), indicative of NMD. Expression of *ltbp3* is not affected by deletion of *ltbp1* (Fig. S2C). Animals homozygous for *ltbp1^fb29^*, including both zygotic and maternal-zygotic mutants, are grossly indistinguishable from siblings during embryogenesis, growth and adulthood (Fig. S2D).

Clutches containing one-quarter DKO embryos were generated by incrossing *ltbp1^−/−^; ltbp3^+/−^* animals. Visual examination revealed that DKO embryos are grossly indistinguishable from siblings prior to 3 dpf, suggesting that zygotic expression of both proteins is dispensable for embryonic development. However, between 3 and 5 dpf, DKO larvae develop a jaw protrusion, mild-pericardial edema (Fig. 1I,J) and aortic regurgitation (Movies 1 and 2). Despite ongoing cardiac contractility, circulation eventually ceases and DKO animals die by ∼8 dpf. To date, we have not identified any adult-viable DKO animals despite genotyping hundreds of adult fish raised from *ltbp1^−/−^; ltbp3^+/−^* incrosses.

To investigate the morphology of DKO hearts, we performed immunostaining for myocardium and Eln2-positive OFT smooth muscle in control and DKO animals at 5 dpf. Remarkably, DKO animals displayed striking enlargements of both the ventricle and OFT, which were readily evident in confocal images (Fig. 1K,L). Examination of single optical sections taken coronally through the center of the heart revealed that the oversized nature of these structures is attributable to aneurysmal expansion of the OFT and dilation of the ventricular chamber (Fig. 1M,N).

### *ltbp1*, *ltbp3* DKO larvae develop rapid ventricular dilation with features of a stress response

**Fig. 2.**
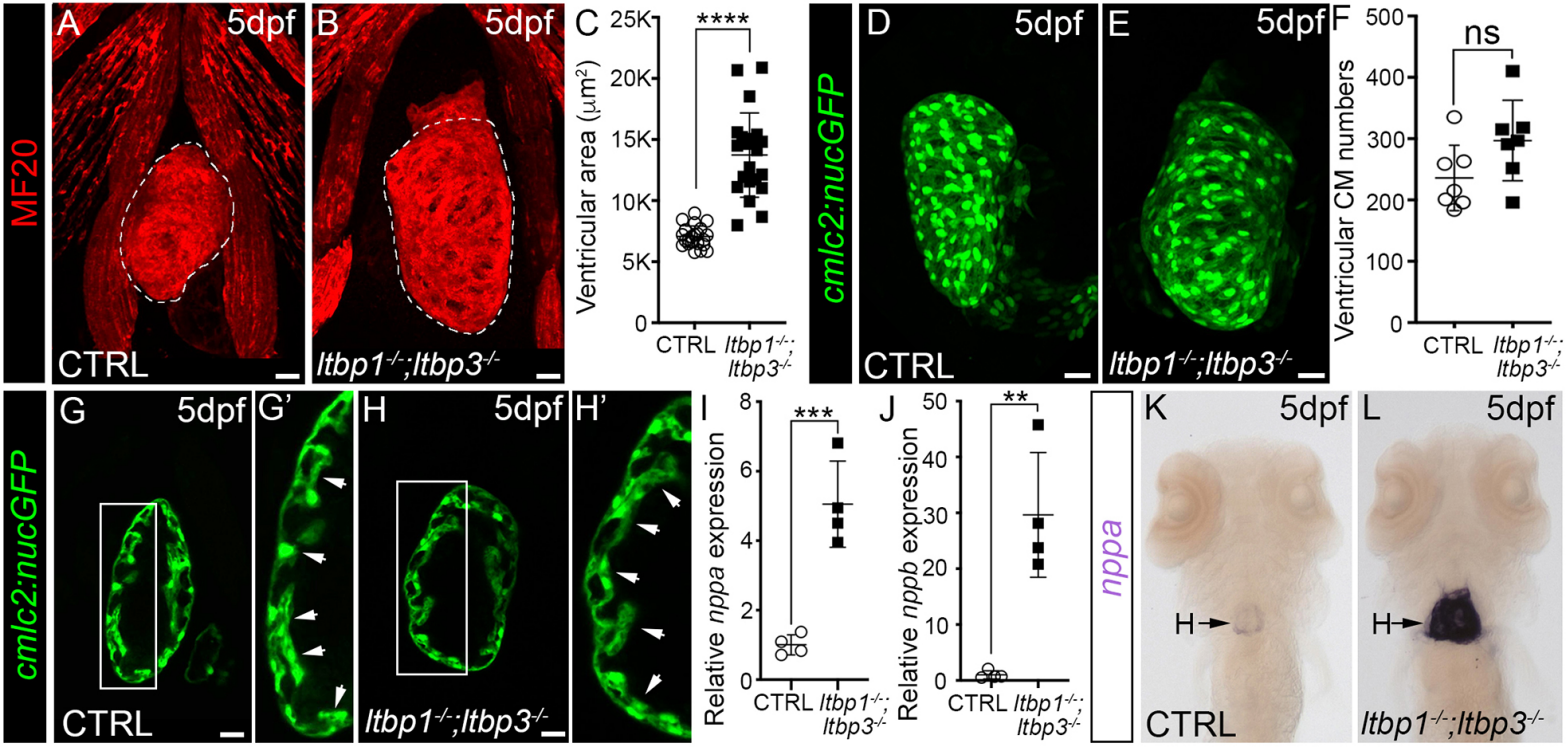
***ltbp1, ltbp3* DKO larvae develop ventricular dilation with molecular features of a stress response.** (A,B) Confocal images of hearts in 5 dpf control (CTRL; A) and *ltbp1^−/−^; ltbp3^−/−^* (B) larvae immunostained for striated muscle (MF20, red). Ventricular size was measured by quantifying the area within the perimeter of the chamber (areas encircled by dashed lines). (C) Dot plot showing the ventricular areas of 5 dpf CTRL (*n*=22) and *ltbp1^−/−^; ltbp3^−/−^* (*n*=21) larvae. (D,E) Confocal images of hearts in 5 dpf CTRL (D) and *ltbp1^−/−^; ltbp3^−/−^* (E) larvae carrying the *cmlc2:nucGFP* transgene and immunostained for GFP. (F) Dot plot showing the number of ventricular cardiomyocytes in 5 dpf CTRL (*n*=7) and *ltbp1^−/−^; ltbp3^−/−^* (*n*=7) larvae. (G-H′) Single optical sections of hearts in 5 dpf CTRL (G,G′) and *ltbp1^−/−^; ltbp3^−/−^* (H,H′) larvae carrying the *cmlc2:nucGFP* transgene and immunostained for GFP. The boxed regions in G and H are shown enlarged in G′ and H′, respectively. Arrowheads highlight trabeculae, observed in seven out of seven ventricles for both experimental groups. (I,J) Dot plots showing the relative expression levels of *nppa* (I) and *nppb* (J) in 5 dpf CTRL and *ltbp1^−/−^; ltbp3^−/−^* larvae measured by qPCR. *n*=4 biological replicates and *n*=3 technical replicates per biological replicate. (K,L) Brightfield images of 5 dpf CTRL (K) and *ltbp1^−/−^; ltbp3^−/−^* (L) larvae processed for whole-mount *in situ* hybridization to detect *nppa* transcripts. Arrows in K and L highlight *nppa* expression in the heart. Little to no variation was observed between animals within each group (*n*>10/group). For all dot plots, statistical significance was determined with an unpaired *t*-test. Error bars show one standard deviation. ****P<0.0001; ***P<0.001; **P<0.01; ns, not significant. Scale bars: 20 µm.

To determine the degree of ventricular dilation, we manually traced chamber perimeters in confocal images of control and DKO hearts (Fig. 2A,B) and quantified the enclosed areas using ImageJ (Materials and Methods). This analysis revealed a twofold increase in ventricular area in DKO animals (Fig. 2C). Even though DKO animals appear grossly unaffected two days earlier on 3 dpf, we confirmed that DKO ventricles do not exhibit chamber dilation at this stage (Fig. S3A-C), demonstrating that ventricular dilation emerges between 3 dpf and 5 dpf. Lastly, we evaluated ventricular areas in single mutants on 5 dpf, which were normal (Fig. S4A-F). Taken together, these data demonstrate that *ltbp1* and *ltbp3* function redundantly to protect the ventricles of early zebrafish larvae from rapid and severe dilation after grossly unperturbed ventricular morphogenesis.

Next, we investigated the cellular basis for ventricular dilation. Beginning on 2 dpf, the zebrafish ventricle grows primarily by cardiomyocyte proliferation (Choi et al., 2013; Pater et al., 2009). To investigate the possibility that chamber dilation stems from expansion of the cardiomyocyte population, we counted ventricular cardiomyocytes on 5 dpf in control and DKO animals carrying a transgene, *cmlc2:nucGFP*, which fluorescently labels cardiomyocyte nuclei (González-Rosa et al., 2018). Control and DKO ventricles contained equivalent numbers of cardiomyocytes (Fig. 2D-F), demonstrating that cardiomyocyte hyperplasia does not account for chamber dilation. Ventricular cardiomyocytes were also unchanged two days prior on 3 dpf (Fig. S3D-F), which further supports the conclusion that ventricular morphogenesis is unperturbed in DKO animals.

During ventricular maturation, cardiomyocytes begin to delaminate from the compact layer on 2 dpf to initiate trabeculation (Liu et al., 2010; Peshkovsky et al., 2011). Impaired delamination would increase the number of cardiomyocytes in the compact layer without affecting total cell number and could potentially lead to chamber dilation. To determine whether DKO hearts exhibit defects in trabeculation, we compared optical sections through the outer curvatures of 5 dpf control and DKO ventricles. Trabeculae were readily evident in both control and DKO animals (Fig. 2G-H′). A qualitative assessment revealed no gross abnormalities in the prevalence or architecture of the trabeculae in DKO ventricles, suggesting that defects in delamination and trabeculation do not account for ventricular dilation. Ultimately, the presence of significant ventricular dilation without elevated cardiomyocyte numbers or defects in trabeculation suggests that ventricular dilation results from a third alternative, which is ventricular cardiomyocyte hypertrophy.

Because *ltbp1* and *ltbp3* are co-expressed in the OFT but not prominently in the ventricle in the two days prior to phenotypic emergence (Fig. 1A-H), we hypothesize that OFT aneurysm represents the primary defect in DKO animals. Under this scenario, the ventricular dilation likely represents pathological chamber remodeling induced by hemodynamic stress associated with OFT distention and aortic regurgitation, which increases preload (Bekeredjian and Grayburn, 2005). To determine whether DKO ventricles express molecular markers of hemodynamic stress, we measured the relative abundance of two cardiomyocyte stress-responsive genes, *nppa* and *nppb* (Becker et al., 2012), by qPCR in control and DKO animals on 5 dpf. Both genes were significantly upregulated in DKO animals (Fig. 2I,J). Using *in situ* hybridization, we localized *nppa* upregulation to the heart (Fig. 2K,L). These data support the hypothesis that pathologic dilation of the DKO ventricle stems from hemodynamic stress.

### *ltbp1*, *ltbp3* DKO larvae develop rapid OFT aneurysm with hyperplastic and hypertrophic features

**Fig. 3.**
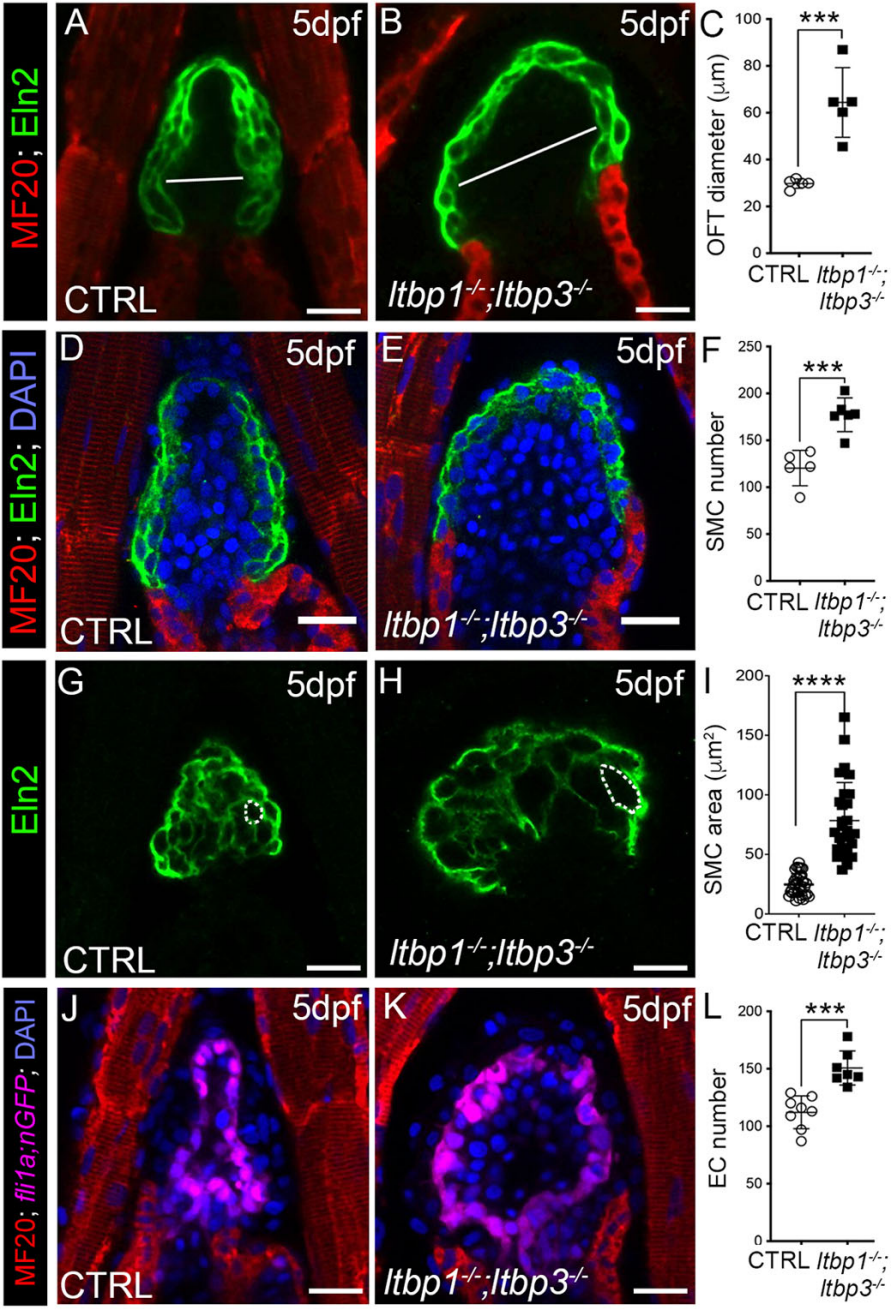
***ltbp1, ltbp3* DKO larvae develop OFT aneurysm with hyperplastic and hypertrophic features.** (A,B,D,E,G,H) Single optical sections through the OFTs of 5 dpf control (CTRL; A,D,G) and *ltbp1^−/−^; ltbp3^−/−^* (B,E,H) larvae double immunostained for striated muslce (A,B; MF20, red) and Eln2-positive OFT smooth muscle (A,B,D,E,G,H; green) and counterstained with DAPI to visualize nuclei (D,E; blue). The white lines in A and B highlight the maximal OFT diameters between the walls of Eln2-positive smooth muscle. DKO OFTs required more optical sections than CTRL OFTs to capture all Eln2-positive cells. (C,F,I) Dot plots showing the maximal OFT diameters, Eln2-positive OFT smooth muscle cell (SMC) numbers or SMC areas in 5 dpf CTRL (*n*=5 in C, *n*=5 in F, *n*=34 total cells from three hearts in I) and *ltbp1^−/−^; ltbp3^−/−^* (*n*=5 in C, *n*=6 in E, *n*=30 total cells from three hearts in I) larvae. SMC number was quantified by counting DAPI-stained nuclei surrounded by Eln2-positive fluorescence. SMC size was measured by quantifying the area within the cell perimeter (areas encircled by dashed lines in G and H). Single optical sections shown in G and H are from *z*-stacks presented in Fig. 1K,L. (J,K) Single optical sections of OFTs from 5 dpf CTRL and *ltbp1^−/−^; ltbp3^−/−^* larvae carrying the endothelial/endocardial *fli1a:nGFP* transgene, double immunostained for striated muscle (MF20, red) and GFP (magenta) and counterstained with DAPI (blue) to visualize nuclei. (L) Dot plot showing the number of endocardial cells in OFTs of 5 dpf CTRL (*n*=8) and *ltbp1^−/−^; ltbp3^−/−^* (*n*=7) larvae. For all dot plots, statistical significance was determined with an unpaired *t*-test. Error bars show one standard deviation. ***P<0.001. ****P<0.0001. Scale bars: 20 µm.

To quantify the degree of OFT dilatation in DKO animals, we compared OFT diameters between 5 dpf control and DKO animals. Similar to the degree of ventricular enlargement, DKO OFTs were twofold larger than those of control animals (Fig. 3A-C). By contrast, single-mutant OFTs were normally sized (Fig. S4G-L), demonstrating that *ltbp1* and *ltbp3* function redundantly to protect the zebrafish OFT from aneurysmal dilatation.

To investigate the cellular basis for OFT aneurysm, we compared several features of the smooth muscle and endocardial cell compartments between control and DKO OFTs on 5 dpf. First, we quantified the number of Eln2-positive smooth muscle cells by counting DAPI-stained nuclei surrounded by fluorescence staining of Eln2. The distended OFTs of DKO animals contained ∼50% more smooth muscle cells (Fig. 3D-F), which was attributable to hyperproliferation based on elevated incorporation of 5-ethynyl-2′-deoxyuridine (EdU) (Fig. S5). Second, we examined mutant OFTs for defects in smooth muscle cell organization. Whereas smooth muscle in control OFTs was composed of 2-3 cell layers that loosely resembled a brick wall (Fig. 3A), DKO OFTs contained fewer cell layers and patterning was erratic (Fig. 3B). Third, we evaluated smooth muscle cell size by quantifying cross-sectional areas in confocal sections (Fig. 3G,H), which revealed a threefold increase in DKO cell size (Fig. 3I). Lastly, we quantified OFT endocardial cells in 5 dpf control and DKO animals carrying a transgene, *fli1:nGFP*, which labels endothelial and endocardial nuclei with GFP (Roman et al., 2002). DKO OFTs contained 34% more endocardial cells than control OFTs (Fig. 3L). Taken together, these data demonstrate that the distended OFTs of DKO animals are composed of hyperplastic endocardial and smooth muscle cell compartments, the latter of which also exhibits patterning defects and cellular hypertrophy.

To determine when these cellular phenotypes emerge, we examined control and DKO OFTs on 3 dpf, before the onset of ventricular dilation and before the OFT becomes visibly expanded in live DKO animals. The OFT diameter (Fig. S6A-C), smooth muscle cell number (Fig. S6D-F) and endocardial cell number (Fig. S6G-I) were all unaltered in DKO animals at this earlier stage. Similarly, smooth muscle cell organization appeared normal in DKO OFTs (Fig. S6A-C). Taken together, these data demonstrate that the OFT phenotypes emerge in DKO animals between 3 and 5 dpf after morphologically unperturbed OFT development.

### Evidence for late hyperactivation of TGFβ signaling in DKO OFTs

Knocking out *ltbp1* and *ltbp3* is predicted to compromise TGFβ signaling events that rely on Ltbp1 and Ltbp3 due to impaired secretion or activation of associated ligands (Rifkin et al., 2018). Similarly, mutations responsible for aneurysm susceptibility in the context of LDS (MacFarlane et al., 2019; Pinard et al., 2019) and, likely, MFS (Cook et al., 2015; Mallat et al., 2017; Rifkin et al., 2018), also undermine TGFβ signaling. Nonetheless, a well-documented molecular feature of syndromic aneurysm tissue from humans and genetically engineered mice is evidence for paradoxical, hyperactivated canonical TGFβ signaling in smooth muscle cells, as determined by immunostaining for phosphorylated SMAD2/3 (pSMAD2/3) (Gomez et al., 2009; Habashi et al., 2006; Holm et al., 2011; Lindsay et al., 2012; Loeys et al., 2005).

**Fig. 4.**
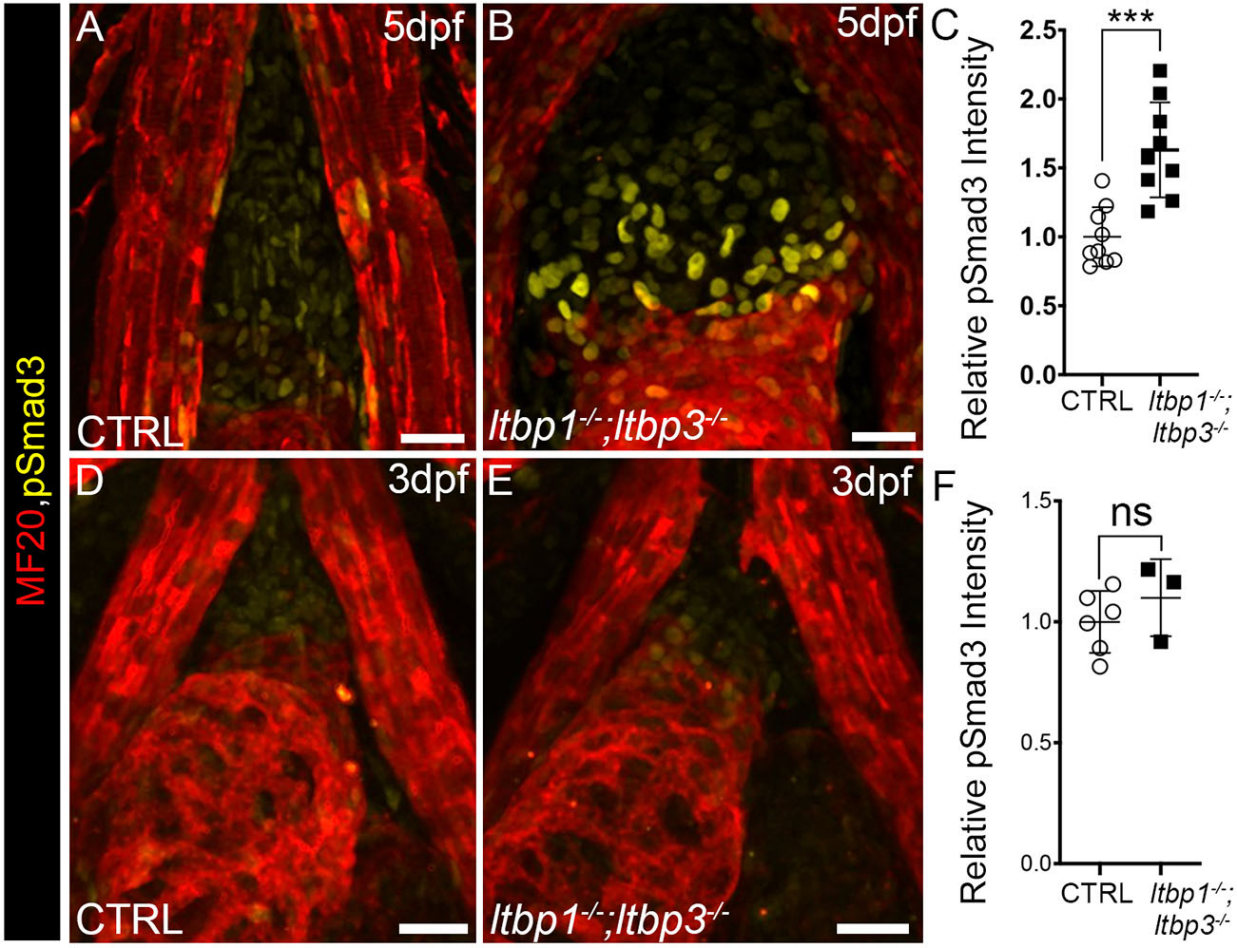
**Hyperactivation of canonical TGFβ signaling in the distended OFTs of *ltbp1, ltbp3* DKO larvae.** (A,B,D,E) Confocal images of OFTs in 5 dpf (A,B) and 3 dpf (D,E) control (CTRL; A,D) and *ltbp1^−/−^; ltbp3^−/−^* (B,E) larvae double immunostained for striated muscle (MF20, red) and phosphorylated Smad3 (pSmad3; green). (C,F) Dot plots showing the relative mean pSmad3 fluorescence intensities in the OFTs of 5 dpf (C) or 3 dpf (F) CTRL (*n*=9 in C; *n*=6 in F) and *ltbp1^−/−^; ltbp3^−/−^* (*n*=9 in C; *n*=3 in F) larvae. For C and F, statistical significance was determined with an unpaired *t*-test. Error bars indicate one standard deviation. ***P<0.01; ns, not significant. Scale bars: 20 µm.

To determine whether the distended OFTs of DKO animals contain elevated pSmad3 levels, we performed immunostaining for pSmad3 on control and DKO animals at 5 dpf. In control animals, modest levels of nuclear pSmad3 signal were detectable in the OFT (Fig. 4A). By comparison, DKO OFTs contained significantly brighter pSmad3-positive nuclei (Fig. 4A,B). Using ImageJ, we quantified mean OFT fluorescence intensities and documented a 63% increase in DKO OFTs (Fig. 4C). Therefore, the distended DKO OFTs recapitulate this molecular hallmark of syndromic aneurysm tissue. We also measured pSmad3 levels on 3dpf, prior to OFT and ventricular expansion, and found them unchanged (Fig. 4D-F), demonstrating that the timing of pSmad3 elevation (3-5 dpf) mirrors that of OFT expansion and ventricular dilation. Finally, to determine the specificity of TGFβ hyperactivation, we tested the hypothesis that signaling events induced by bone morphogenetic proteins (BMPs), i.e. additional members of the TGFβ superfamily, are also hyperactivated in DKO OFTs. To that end, we performed immunostaining for the BMP effectors phosphorylated Smad1/5/9(8) (Derynck and Budi, 2019) and did not observe evidence for BMP hyperactivation (Fig. S7).

**Fig. 5.**
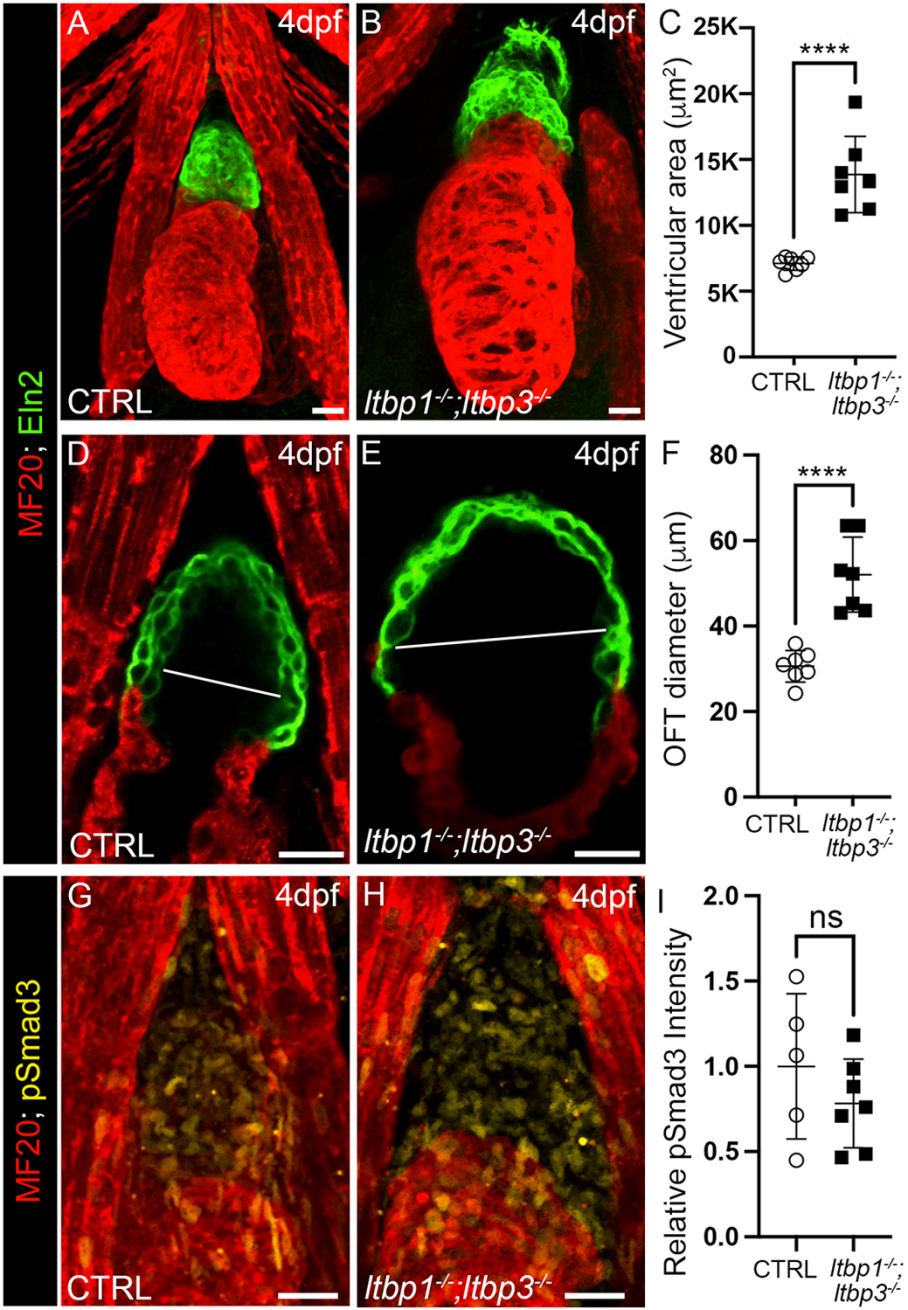
**OFT aneurysm and ventricular dilation precede hyperactivation of TGFβ signaling in *ltbp1, ltbp3* DKO larvae.** (A,B) Confocal images of hearts in 4 dpf control (CTRL; A) and *ltbp1^−/−^; ltbp3^−/−^* (B) larvae double immunostained for striated muscle (MF20, red) and Eln2-positive OFT smooth muscle (green). (C) Dot plot showing the ventricular areas of 4 dpf CTRL (*n*=7) and *ltbp1^−/−^; ltbp3^−/−^* (*n*=7) larvae. (D,E) Single optical sections through the OFTs of 4 dpf CTRL (D) and *ltbp1^−/−^; ltbp3^−/−^* (E) larvae double immunostained for striated muscle (MF20, red) and Eln2-positive OFT smooth muscle (green). The white lines highlight the maximal OFT diameters between the walls of Eln2-positive smooth muscle. (F) Dot plot showing the maximal OFT diameters in 4 dpf CTRL (*n*=7) and *ltbp1^−/−^; ltbp3^−/−^* larvae (*n*=7). (G,H) Confocal images of OFTs in 4 dpf CTRL (G) and *ltbp1^−/−^; ltbp3^−/−^* (H) larvae double immunostained for striated muscle (MF20, red) and phosphorylated Smad3 (pSmad3, green). Dot plot showing the relative mean pSmad3 fluorescence intensities in the OFTs of 4 dpf CTRL (*n*=5) and *ltbp1^−/−^; ltbp3^−/−^* (*n*=7) larvae. For all dot plots, statistical significance was determined with an unpaired *t*-test. Error bars indicate one standard deviation. ****P<0.0001. ns, not significant. Scale bars: 20 µm.

Next, we more thoroughly investigated the temporal relationship between elevated pSmad3, OFT aneurysm and ventricular dilation. To refine the developmental window during which the ventricular and OFT phenotypes emerge, we analyzed DKO animals on 4 dpf and learned that DKO animals were already displaying phenotypes at this intermediate stage (Fig. 5A-F). Analysis of pSmad3 levels at 4 dpf however, revealed no difference from controls (Fig. 5G-I), demonstrating that enlargement of the ventricle and OFT in DKO animals precedes hyperactivation of TGFβ signaling and not vice versa. These data suggest that hyperactivated TGFβ signaling in DKO animals is not a driver of OFT aneurysm or ventricular dilation but, rather, a downstream consequence of disease pathogenesis or progression.

### Similarities between the molecular signatures of disease tissue from DKO animals and MFS mice

**Fig. 6.**
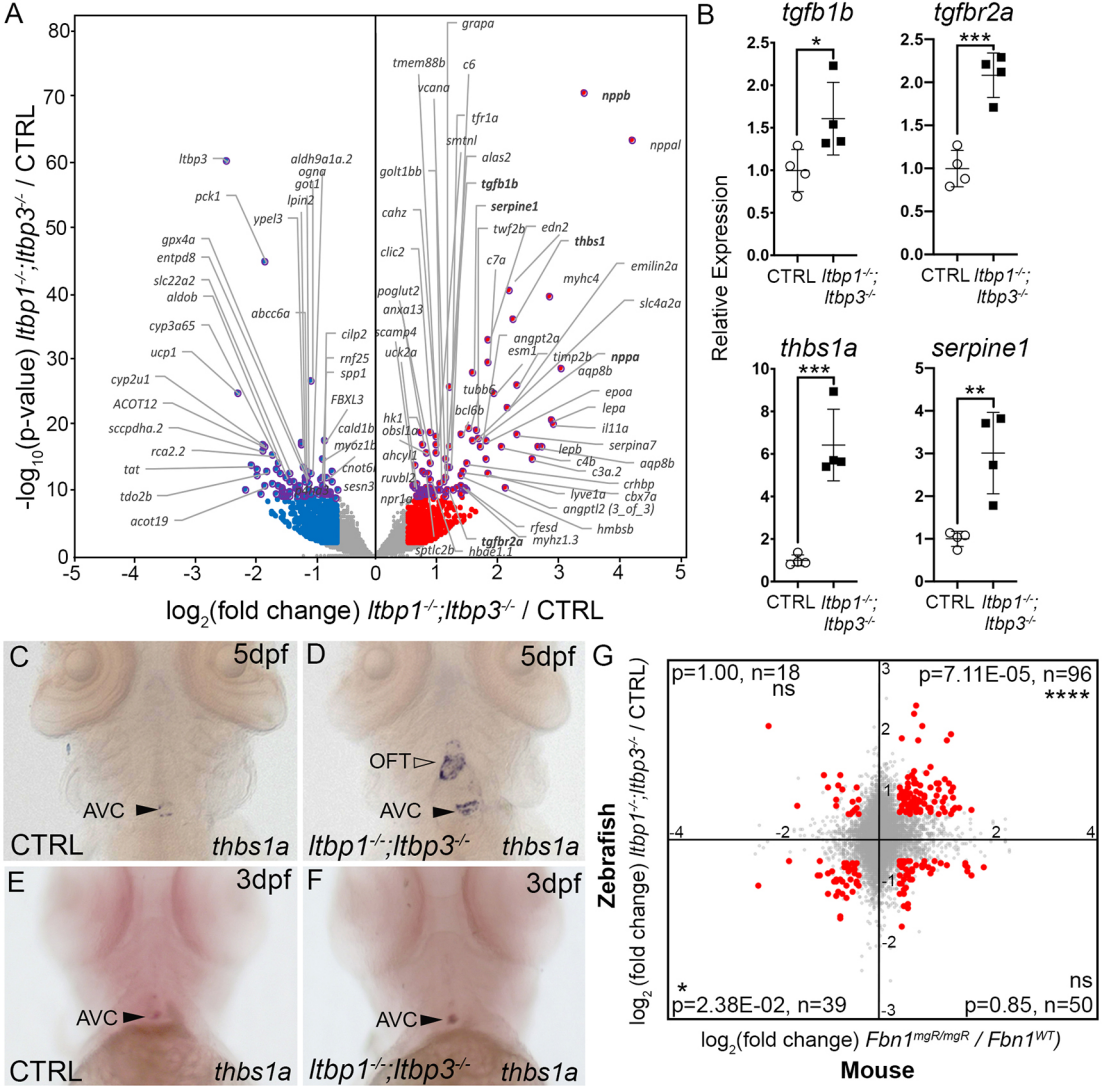
**Molecular similarities between disease tissue from *ltbp1, ltbp3* DKO larvae and ascending aortic aneurysms from a mouse model of Marfan syndrome.** (A) Volcano plot showing the distribution of log_2_-fold changes and raw *P*-values for protein-coding RNAs isolated from co-dissected OFTs and ventricles of 5 dpf *ltbp1^−/−^; ltbp3^−/−^* larvae relative to control (CTRL) samples. Genes with raw *P*<10^−10^ are highlighted in purple and official gene symbols are provided. Genes meeting the inclusion criteria for GO term enrichment analysis – i.e. |log_2_-fold change|>0.58496, equivalent to |fold change|>1.5, and adjusted *P*<0.05 – are plotted in purple and red (upregulated) or blue (downregulated). Gene symbols in bold highlight transcriptional alterations consistent with hyperactivated TGFβ signaling or cardiac stress. (B) Dot plots showing the relative expression levels of *tgfb1b, tgfbr2a, thbs1a* and *serpine1* transcripts in 5 dpf CTRL and *ltbp1^−/−^; ltbp3^−/−^* larvae as determined by qPCR. Statistical significance was determined with an unpaired *t*-test. *n*=4 biological replicates and *n*=3 technical replicates. Errors bars show one standard deviation. *P<0.05; **P<0.01; ***P<0.001. (C-F) Brightfield images of 5 dpf (C,D) and 3 dpf (E,F) CTRL (C,E) and *ltbp1^−/−^; ltbp3^−/−^* (D,F) larvae processed for whole-mount *in situ* hybridization to detect *thbs1a* transcripts. Closed and open arrowheads highlight the atrioventricular canal (AVC) and outflow tract (OFT), respectively. Little to no variation was observed between animals within each group (*n*>10/group). (G) Coordinate plane showing the log_2_-fold changes as in A plotted on the *y*-axis, and the log_2_-fold changes for orthologous mouse genes in aneurysm tissue from *Fbn1^mgR/mgR^* mice relative to control tissue plotted on the *x*-axis. Mouse data were sourced from Zilberberg et al. (2015). Orthologous gene pairs meeting the inclusion criteria for GO-term enrichment analysis – i.e. |log_2_-fold change|>0.37851, equivalent to |fold change|>1.3, and adjusted *P*<0.1 in both datasets – are highlighted in red. For each quadrant, the probability (p) that the indicated number of gene pairs (*n*) fulfills the inclusion criteria by chance was determined using a hypergeometric test. ns, not significant. ****P<0.0001, *P<0.05.

To characterize the molecular alterations in DKO hearts on a global scale, we performed RNA-sequencing of co-dissected ventricles and OFTs from 5 dpf control and DKO animals. Owing to the small size of the zebrafish heart, manual separation of the OFT from the ventricle to obtain structure-specific molecular signatures is not feasible. This analysis revealed upregulation and downregulation of 810 and 961 protein-coding transcripts, respectively (|FC|>1.5, adjusted *P*<0.05; Fig. 6A; Table S1). Gene ontology (GO) term enrichment analysis of the upregulated gene set identified ‘protein folding’ as the top functional category likely reflecting, at least partially, an unfolded protein response associated with cellular stress (Fig. S8A; Table S2). Accordingly, GO term enrichment analysis also identified ‘unfolded protein binding’ just below significance (adjusted *P*=0.059; Fig. S8A). ‘Protein folding’ could also be interpreted in the context of other categories, including ‘ribosome biogenesis in eukaryotes’, ‘AA-tRNA biosynthesis’, ‘mitochondrion’ and ‘rRNA processing’, to suggest that DKO cells produce nascent proteins and ATP at higher rates due to cellular hypertrophy. In the downregulated gene set, GO term enrichment analysis identified the terms ‘metabolic process’, ‘oxidoreductase activity’, ‘biosynthesis of amino acids’ and ‘gluconeogenesis’ (Fig. 8A), suggesting that DKO cells suffer from metabolic abnormalities as a cause and/or consequence of cellular stress. We also performed gene set enrichment analysis (GSEA) on the human orthologs of the differentially expressed zebrafish genes and retrieved similar categories but also an indication of increased cell cycle activity in DKO animals (Table S3), consistent with the observed OFT hyperplasia (Fig. 3D-F,J-L; Fig. S5).

A targeted search in the dataset for transcriptional alterations related to TGFβ signaling revealed DKO upregulation of the ligand *transforming growth factor beta 1b* (*tgfb1b*), *transforming growth factor beta receptor 2a* (*tgfbr2a*), an activator of latent TGFβ complexes *thrombospondin1a* (*thbs1a*) (Crawford et al., 1998) and the TGFβ target gene *serpine1* (also known as pai-1) (Dennler et al., 1998; Hua et al., 1998). These molecular changes were confirmed by qPCR (Fig. 6B) and are consistent with elevated pSmad3 levels and hyperactivation of TGFβ signaling in the expanded OFTs of DKO animals. Using *in situ* hybridization, we confirmed that *thbs1a* upregulation is localized to the DKO OFT (Fig. 6C,D). However, similar to pSmad3, *thbs1a* was not upregulated prior to OFT aneurysm (Fig. 6E,F), suggesting that *thbs1a* upregulation is not driving the phenotype. Also notable in the RNA-sequencing dataset was the increased expression of *angiotensin II type receptor type I* (*agtr1b*) (fold change=2.079, adjusted *P*=7.95e-05), the target of the widely prescribed drug losartan for slowing aneurysm progression (Bowman et al., 2019).

To determine whether the molecular alterations observed in disease tissue from DKO animals showed similarities to those in aneurysm tissue from a mouse model of MFS, we cross-referenced our dataset with a previously published microarray analysis of ascending aortic aneurysms from *Fbn1^mgR/mgR^* mice (Zilberberg et al., 2015). By comparing the most upregulated or downregulated genes in both datasets (|fold change| >1.3, adjusted *P*<0.1), we found that several orthologous gene pairs are jointly up- or downregulated in both settings. This occurred at frequencies that were higher than would be predicted to occur by chance (Fig. 6G; Table S4), suggesting that disease tissue from DKO animals and MFS mice shares a molecular signature. GO term enrichment analysis identified functional categories in the jointly upregulated orthology pairs that are consistent with immune/blood-cell infiltration (i.e. complement and coagulation cascades, immunity, inflammatory response, hematopoietic cell lineages), modified cell-cell and cell-ECM adhesion/interactions (i.e. cell adhesion, signal peptide, integrin complex), alterations in ECM composition (i.e. glycoprotein) and cell cycle activity (i.e. cell division, cell cycle) (Fig. S8B; Table S5). No functional categories were significantly enriched in the jointly downregulated orthology pairs (Table S5).

### TGFβ signaling is protective against OFT aneurysm and ventricular dilation in larval zebrafish

Given that knocking out *ltbp1* and *ltbp3* has been predicted to undermine those TGFβ signaling events that rely on Ltbp1 and Ltbp3 (Rifkin et al., 2018), we hypothesized that OFT aneurysm in DKO animals is due to impaired TGFβ signaling. At first, this appears paradoxical given the evidence of TGFβ hyperactivation in the overtly dilated OFTs (Fig. 5A-C). However, as detailed above, this paradox is well documented in syndromic aneurysm but also remains unresolved on molecular level. Moreover, hyperactivation of TGFβ signaling follows, rather than precedes, OFT aneurysm and ventricular dilation (Fig. 5), suggesting that it is secondary, rather than primary.

**Fig. 7.**
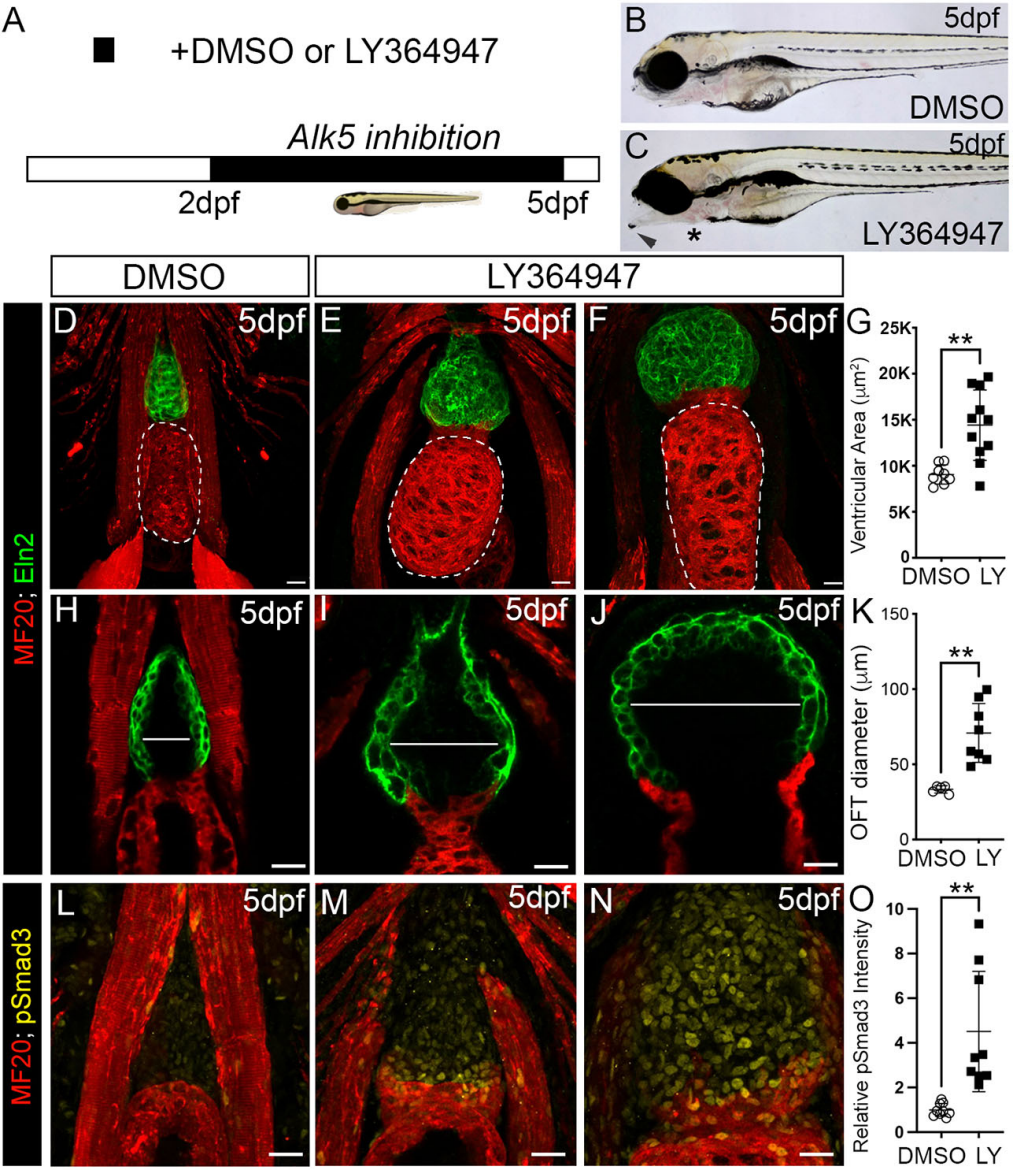
**TGFβ signaling protects larval zebrafish from OFT aneurysm and ventricular dilation.** (A) Experimental timeline for small-molecule-mediated inhibition of TGFβ signaling in wild-type animals. (B,C) Brightfield images of 5 dpf wild-type larvae treated with DMSO (B) or LY364947 (C). Arrowhead and * in C highlight a jaw protrusion and mild pericardial edema, respectively, observed in LY364947-treated animals. (D-F) Confocal images of hearts in 5 dpf wild-type animals treated with DMSO (D) or LY364947 (E,F) and double immunostained for striated muscle (MF20, red) and Eln2-positive OFT smooth muscle (green). E and F show representative hearts with moderate and severe phenotypes, respectively. Ventricular size was measured by quantifying the area within the perimeter of the chamber (areas encircled by dashed lines in D-F). (G) Dot plot showing the ventricular areas of 5 dpf wild-type larvae treated with DMSO (*n*=8) or LY364947 (*n*=11). (H-J) Single optical sections taken from the images shown in D-F through the OFTs of 5 dpf wild-type larvae treated with DMSO (H) or LY364947 (I,J). White lines show the maximal OFT diameters between the Eln2-positive walls of smooth muscle. (K) Dot plot showing the OFT diameters in DMSO-(*n*=5) and LY364947-(*n*=8) treated animals. (L-N) Confocal images of OFTs in 5 dpf wild-type animals treated with DMSO (L) or LY364947 (M,N) double immunostained for striated muscle (MF20, red) and phosphorylated Smad3 (pSmad3; green). (O) Dot plot showing the relative mean pSmad3 fluorescence intensities in the OFTs of DMSO-(*n*=9) or LY364947-(*n*=9) treated larvae. For all dot plots, statistical significance was determined with an unpaired *t*-test. Error bars show one standard deviation. **P<0.01. Scale bars: 20 µm.

If OFT aneurysm in DKO animals stems from an impairment of TGFβ signaling, then inhibition of TGFβ signaling in wild-type animals should phenocopy DKO animals. Therefore, we evaluated wild-type animals after exposure to the TGFβ antagonist LY364947, which competes with ATP for binding to the kinase domain of the TGFβ type I receptor and prevents phosphorylation of Smad2/3 (Peng et al., 2005; Sawyer et al., 2003). First, we validated LY364947 by treating 48 hpf wild-type embryos with DMSO or LY364947 for 4 h (Fig. S9A) before immunostaining with the pSmad3 antibody. As expected, LY364947-treated embryos displayed a significant reduction in pSmad3 levels in the OFT (Fig. S9B-D). Next, we exposed wild-type animals to LY364947 or DMSO on 2-5 dpf (Fig. 7A) and measured ventricular areas, OFT diameters and pSmad3 fluorescence intensities. Similar to DKO animals, LY364947-treated animals developed a jaw protrusion and mild pericardial edema (Fig. 7B,C). Quantification of ventricular areas and OFT diameters revealed significant enlargements of both structures ranging from moderate (Fig. 7D,E,H,I) to severe (Fig. 7D,F,H,J), with averages similar to those observed in DKO animals (Figs 7G,K, 2A-C, 3A-C). Moreover, as in DKO animals, pSmad3 levels were significantly elevated in the distended OFTs of DMSO- or LY364947-treated animals (Fig. 7L-O). Taken together, these data demonstrate that inhibition of TGFβ in wild-type animals is sufficient to phenocopy DKO animals. They also indicate that hyperactivation of TGFβ signaling in the dilated OFT, which occurs as a downstream consequence of TGFβ inhibition, is an embodiment of the same aforementioned paradox observed in syndromic aneurysm tissue. Lastly, given the evidence for late hyperactivation of TGFβ signaling in DKO animals, we treated double mutants with LY364947 on 2-5 dpf (Fig. S10A) and measured OFT diameters, which were not significantly different from those in DMSO-treated DKO animals (Fig. S10B-D), demonstrating that, in this context, suppression of TGFβ signaling does not rescue the OFT phenotype.

To determine when TGFβ signaling is required to prevent OFT aneurysm, we treated wild-type animals on 2-3 dpf (Fig. S11A), a developmental window that overlaps with co-expression of *ltbp3* and *ltbp1* in the OFT but precedes OFT aneurysm in DKO animals. We also treated wild-type animals on 4-5 dpf (Fig. S11E), a developmental window subsequent to *ltbp3* and *ltbp1* co-expression, which coincides temporally with OFT aneurysm in DKO animals. Whereas exposure on 2-3 dpf failed to expand the OFT diameter (Fig. S11B-D), treatment on 4-5 dpf successfully induced aneurysm (Fig. S11F-H). The observation that TGFβ signaling is required after co-expression of *ltbp1* and *ltbp3* is consistent with the molecular function of LTBP proteins. Specifically, they anchor latent TGFβ complexes to the ECM until signaling becomes activated through ligand release (Rifkin et al., 2018). Therefore, differences can exist between the timing of *LTBP* expression and downstream TGFβ signaling.

Lastly, given that knocking out *ltbp1* and *ltbp3* has been predicted to lower TGFβ signaling (Rifkin et al., 2018) and that inhibition of TGFβ is sufficient to phenocopy DKO animals, we attempted to rescue DKO animals by treating them with SRI-011381, a small-molecule agonist of TGFβ signaling (Liu et al., 2018). First, we validated SRI-011381 by exposing wild-type animals to DMSO or SRI-011381 on 2-5 dpf (Fig. S12A) and documenting a 70% increase in pSmad3 levels in the OFTs of SRI-011381-treated animals (Fig. S12B-D). SRI-011381-treated wild-type animals did not exhibit OFT expansion (Fig. S13A-D), suggesting that hyperactivation of TGFβ signaling is not sufficient to induce aneurysm. Similarly, treating DKO animals on 2-5 dpf (Fig. S13A) with SRI-011381 did not alter OFT diameter compared to DMSO-treated controls (Fig. S13E-G), demonstrating that hyperactivation of TGFβ signaling also does not suppress aneurysm in DKO animals.

## DISCUSSION

Our data demonstrate that the TGFβ regulatory proteins Ltbp1 and Ltbp3 function redundantly to protect the zebrafish OFT, which is equivalent to the aortic root in humans, from rapid and severe aneurysmal dilatation. In DKO animals, the OFT diameter rapidly doubles in size following morphologically unperturbed OFT development. During the same time window, the ventricle also dilates significantly following grossly normal ventricular morphogenesis. Given that *ltbp1* and *ltbp3* are co-expressed in the OFT prior to phenotypic emergence, albeit not measurably in the ventricle, OFT aneurysm in DKO animals is almost certainly a primary phenotype. From our data, we cannot determine whether the ventricular dilation is also a primary phenotype or a secondary consequence of aortic regurgitation. Indeed, aortic regurgitation has been cited to be a contributing factor to help to explain why some MFS patients and mouse models are vulnerable to dilated cardiomyopathy and heart failure (Cook et al., 2014). However, mounting evidence suggests that pathologic remodeling might also be a direct consequence of compromised *FBN1* function in the myocardium (Alpendurada et al., 2010; Cook et al., 2014; Rouf et al., 2017; Tae et al., 2016). Although dilated cardiomyopathy is not a prominent feature of LDS or other inherited forms of TAAs (Guo et al., 2007; Loeys et al., 2005; Zhu et al., 2006), the rapidity of OFT expansion in DKO animals might explain the heightened susceptibility in this context. Theoretically, it remains possible that ventricular dilation is also a primary phenotype, since we cannot rule out the possibility that *ltbp1* and *ltbp3* are coexpressed in the ventricle below levels of detection where they actively protect the ventricle from dilation. It also remains possible that deposition of Ltbp3- and Ltbp1-containing complexes in the ventricular wall before 48 hpf provides protection at later stages*.* Indeed, we have previously documented *ltbp3* expression in ventricular cardiomyocytes of the linear heart tube and in SHF progenitors that give rise to the distal ventricular myocardium prior to 48 hpf (Zhou et al., 2011). Uncovering the tissue-specific requirements for *ltbp1* and *ltbp3* would require conditionally knocking out either gene in the ventricular myocardium or OFT, which is currently not feasible without floxed alleles. Tissue-specific re-expression of *ltbp1* or *ltbp3* in DKO animals could also distinguish primary from secondary phenotypes.

Many similarities exist between the distended OFTs of *ltbp1^−/−^; ltbp3^−/−^* zebrafish and aortic root aneurysms in human. Specifically, the OFT diameter enlarges by 100%, which is above the clinical threshold (50%) for diagnosing aortic aneurysm (Hiratzka et al., 2010). The smooth muscle in the distended OFTs is hypercellular and disorganized, which has been documented in aneurysm samples from humans (Guo et al., 2007; Pannu et al., 2007; Tang et al., 2005). The dilated OFTs show evidence of elevated canonical TGFβ signaling, a prominent feature of aneurysm tissue in MFS and LDS patients and mouse models (Gomez et al., 2009; Habashi et al., 2006; Holm et al., 2011; Lindsay et al., 2012; Loeys et al., 2005). Lastly, cardiac tissue from mutant animals displays a molecular signature that significantly overlaps with aneurysm tissue from a mouse model of MFS.

One well-described hallmark of TAAs in humans and mouse models is fragmentation of elastic fibers in the tunica media (Habashi et al., 2006; Hiratzka et al., 2010; Lindsay et al., 2012). Although elastic fibers appear to be present in the OFTs of adult zebrafish (Hu et al., 2001), we did not observe thick extracellular Eln2-positive fibers in confocal images of immunostained wild-type larvae at the developmental stages analyzed. Of note, however, elastic fiber fragmentation is not considered a significant driver of aneurysm pathogenesis (Lindsay and Dietz, 2014). The most serious consequence of aneurysm is aortic dissection (Hiratzka et al., 2010). During the later stages of the DKO phenotype, circulation ceases altogether, even though the heart continues to beat – consistent with the possibility that, similar to aortic dissection, a disrupted endocardial layer obstructs blood efflux from the OFT.

Our data suggest that TGFβ signaling protects the larval zebrafish OFT from aneurysmal dilation. This conclusion is based on the presence of OFT expansion in wild-type animals treated with a small-molecule inhibitor of the TGFβ type I receptor, e.g. LY364947. It is also based on the documented OFT aneurysm in DKO animals where TGFβ signaling was predicted to be compromised, based on prior knowledge of LTBP function (Rifkin et al., 2018). Specifically, in the absence of an LTBP protein, the TGFβ propeptide becomes susceptible to proteolysis prior to secretion. Also, in the unlikely event that some SLC complexes were to be secreted in DKO animals, the absence of any physical association with the ECM – otherwise mediated by LTBPs – is likely to preclude integrin-mediated ligand activation. Despite speculation that TGFβ signaling is reduced in DKO animals, we have not uncovered experimental evidence for low TGFβ signaling in DKO animals. Moreover, chemical activation of TGFβ signaling did not rescue the DKO phenotype. Perhaps any decreases in signaling in DKO animals are sufficiently transient or subtle to evade detection. Similarly, the manner in which we were activating TGFβ signaling might not recapitulate the timing, magnitude or lineage specificity of signaling, which otherwise protects the OFT from aneurysm in wild-type animals. Lastly, it remains possible that Ltbp1 and Ltbp3 are performing molecular functions that are independent of TGFβ ligand regulation (Guo et al., 2018; Rifkin et al., 2018). However, we find this less likely, given the near-perfect phenocopy between DKO and wild-type animals treated with the TGFβ inhibitor LY364947.

While this study was in progress, another study also implicated TGFβ signaling in protecting the zebrafish OFT from aneurysmal expansion (Boezio et al., 2020). The authors generated and characterized zebrafish embryos devoid of the TGFβ type I receptor Alk5. Like DKO animals, *alk5* mutants exhibit OFT expansion associated with endocardial hyperplasia. Unlike DKO animals, however, OFT smooth muscle cells in *alk5* mutants are less proliferative and lower in number. The timing of OFT expansion also differs. Whereas the OFT phenotype in *alk5* mutants emerges between 24 hpf and 78 hpf, it emerges much later in DKO animals, i.e. between 72 hpf and 120 hpf (3-5 dpf) after grossly unperturbed OFT development. Moreover, although DKO animals exhibit significant ventricular dilation, the ventricular chamber in *alk5* animals is unaltered at 78 hpf. Based on expression profiling and rescue studies, the phenotypes in *alk5* mutants were, in part, attributable to decreased expression of the ECM gene *fibulin 5* in the endothelium of the OFT or aortic arch I. Fibulin 5 was not significantly decreased in our differential expression analysis (Table S1). The differences in phenotype likely reflect tissue- or stage-specific roles played by Alk5 or Ltbp1/3 in TGFβ-mediated OFT development or homeostasis. Additionally, whereas *ltbp1* and *ltbp3* mutations were predicted to compromise only those TGFβ signaling events normally facilitated by Ltbp1 and Ltbp3, *alk5* mutants should be completely devoid of all TGFβ signaling, another factor likely contributing to phenotypic differences.

The conclusion that baseline TGFβ signaling is protective against aortic aneurysm is supported by mouse studies, in which homozygous deletions of TGFBRI or TGFBRII in aortic smooth muscle have been reported to be sufficient for initiating TAAs (Choudhary et al., 2009; Hu et al., 2015; Li et al., 2014; Schmit et al., 2015). The relevance of these mouse models to human disease was questioned, however, because the aneurysms emerge over a significantly accelerated time frame (MacFarlane et al., 2019). While the rapid pace of OFT dilatation in DKO animals would certainly be subject to the same criticism, studying an early and rapidly emerging phenotype with disease features may have some advantages. Particularly, an unbiased small-molecule suppressor screen becomes feasible since, by 5 dpf, the aneurysm phenotype has 100% penetrance and animals at this stage are small enough to allow screening in a microwell format.

Our study also suggests that the observed TGFβ hyperactivation is not driving OFT aneurysm in DKO animals. We base this conclusion on the failure of experimental hyperactivation of TGFβ signaling to induce aneurysm in wild-type animals and on the temporal relationship between OFT expansion and hyperactivation of TGFβ signaling in that the former precedes the latter. The paradoxical hyperactivation of TGFβ signaling likely reflects a compensatory response to insufficient TGFβ signaling or a non-specific reaction to vessel wall stress, as postulated for syndromic aneurysm (Cook et al., 2015; Holm et al., 2011; Lindsay and Dietz, 2011; MacFarlane et al., 2019; Mallat et al., 2017; Milewicz et al., 2017; Rifkin et al., 2018).

The generation and phenotypic analysis of *ltbp3* null embryos undermines our previous conclusion that *ltbp3* is required for SHF development in zebrafish, which was based on morpholino-mediated knockdown studies (Zhou et al., 2011). Whereas *ltbp3* morphants suffer severe reduction of SHF-derived ventricular cardiomyocytes at 48 hpf (Zhou et al., 2011), *ltbp3* null ventricles have normal cardiomyocyte numbers at this stage. Moreover, whereas *ltbp3* morphants lack SHF-derived Eln2-positive OFT smooth muscle at 72 hpf (Zhou et al., 2011), OFT smooth muscle cell numbers are normal at this stage in DKO animals. The potential for discordance between morphant and mutant phenotypes is well documented (Kok et al., 2015) and can be explained by genetic compensation in some (El-Brolosy et al., 2019; Ma et al., 2019) but not all cases (Tessadori et al., 2020). Although *ltbp1* expression levels are not increased in *ltbp3* mutants, we tested the hypothesis that *ltbp1* and *ltbp3* are redundant, by generating and analyzing DKO animals. Given that DKO animals show the normal numbers of ventricular cardiomyocytes and OFT smooth muscle cells at 3 dpf, SHF defects are not readily evident. Therefore, whereas it certainly remains possible that alternative genes are responsible for compensation in *ltbp3* null animals, we favor the simpler explanation that morpholino-mediated off-target effects or toxicity induced the SHF phenotype we documented previously in *ltbp3* morphants (Zhou et al., 2011). If this is true, then *ltbp3* is, indeed, dispensable for SHF development in zebrafish, a conclusion that is consistent with the lack of congenital heart defects in *ltbp3* knockout mice (Dabovic et al., 2002).

Mutations in *LTBP3* and deletions encompassing the *LTBP1* locus have recently been linked to TAAs in humans. In 2018, Guo and colleagues reported that pathogenic variants in *LTBP3* segregated with aneurysms of the aortic root and/or ascending aorta and aortic dissections in two families (Guo et al., 2018). In the same study, a reevaluation of *LTBP3* knockout mice, which had originally been reported to have normally sized aortic roots and ascending aortas (Zilberberg et al., 2015), revealed expansion of both after taking into account the diminutive size of the null animals (Guo et al., 2018). Also in 2018, a heterozygous 5.1Mb deletion involving 11 genes, including *LTBP1*, was reported to segregate with aortic root and/or ascending aortic dilation within a single family (Quiñones-Pérez et al., 2018). Given the known association between perturbations in TGFβ signaling and TAAs, the authors speculate that deletion of the *LTBP1* gene is causal, or at least a contributory factor, for disease pathogenesis in this family. Despite this possibility, a more-recent study described eight individuals aged between 1.5 and 17 years carrying bi-allelic truncating variants in *LTBP1* (Pottie et al., 2021) and none was reported to suffer from aneurysm. *LTBP1* knockout mice have been studied extensively but, because they die during the perinatal period, assessment of aortic root diameter thereafter is not possible (Horiguchi et al., 2015; Todorovic et al., 2007, 2011). In summary, although mutations in *LTBP3* have been linked to human aneurysmal disease, the causality of deletions involving the *LTBP1* locus remains speculative.

Defects in the smooth muscle cell elastin-contractile unit have been put forth as a unifying model to explain how mutations in three groups of proteins, including TGFβ signaling components, ECM components and smooth muscle contractile proteins, all lead to TAAs (Pinard et al., 2019). How might the absence of Ltbp3 and Ltbp1 undermine the contractility of this unit? TGFβ signaling is known to play a crucial role in promoting smooth muscle cell differentiation and a contractile phenotype (Milewicz et al., 2017; Rensen et al., 2007). Therefore, with lowered or absent TGFβ signaling, smooth muscle cells are likely to remain under-differentiated and hyperproliferative, the latter of which is evident by the increasing numbers of smooth muscle cells in DKO animals. Future studies, including those incorporating small-molecule suppressor screens, will provide additional insights into the molecular pathogenesis of OFT aneurysm in DKO animals.

## MATERIALS AND METHODS

### Zebrafish husbandry and strains

Zebrafish were produced, grown and maintained according to protocols approved by the Massachusetts General Hospital Institutional Animal Care and Use Committee. Wild-type animals and those carrying the following alleles and transgenes were utilized: *ltbp3^fb28^* (this study), *cmlc2:nucGFP* (González-Rosa et al., 2018), *ltbp1^fb29^* (this study) and *Tg(fli1a:nEGFP)^y7^* (Roman et al., 2002). For all experiments involving null animals, sibling animals of all genotypes were used as controls.

### Generation and detection of *ltbp3^fb28^* and *ltbp1^fb29^* alleles

The domain structures of zebrafish Ltbp3 (UniProtKB: F1QFX6) and Ltbp1 (UniProtKB:F1QQ56) were reproduced from the InterPro database (Mitchell et al., 2019), with the exception of the hybrid domains, which were identified based on alignments with the human homologs (Jensen et al., 2009). A pair of TALENs targeting exon 3 of zebrafish *ltbp3* was designed and generated as described (Ma et al., 2013). The variable di-residue (RVD) sequences were: HD NG NN NG NN NG HD NI HD NG HD NG NN HD and NI HD NG HD NI NN NI NN NI NN NG. mRNAs encoding each TALEN were produced and co-injected into one-cell stage zebrafish embryos as described (Ma et al., 2013). A guide RNA targeting the sequence 5′-GGATGCCTGTTGTGGGACGGTGG-3′ in exon 14 of the *ltbp1* locus (*ltbp1*-201; ENSDART00000079460.6) was generated and co-injected with Cas9 mRNA into one-cell stage zebrafish embryos as described (Jao et al., 2013). Germline transmission of TALEN- and CRISPR/Cas9-induced mutations was detected using fluorescent PCR and DNA-fragment analysis as described (Foley et al., 2009). The *ltbp3^fb28^* allele lacks seven base pairs, delCTGGACA, in exon 3 of *ltbp3*. The *ltbp1^fb29^* allele lacks eight base pairs, delACGGTGGG, in exon 14 of *ltbp1*. Primers used to distinguish wild-type from mutant alleles of *ltbp3* by fluorescent PCR and DNA-fragment analysis were forward 5′-CTCAAGCTACTCGTGGCAACAAGCA-3′ and reverse 6-FAM 5′-TGAGTTTGACACCCCTGCTTTAGATTG-3′, yielding amplicons of 465 bp for the wild-type allele and of 458 bp for the mutant allele. Primers used to distinguish wild-type from mutant alleles of *ltbp1* by fluorescent PCR were forward 5′-GCTGTGCCTATTTGTGCAAC-3′ and reverse 6-FAM 5′-TCATGAGAGTGCATCAACAGC-3′, yielding amplicons of 437 or 441 bp for the wild-type allele and of 429 bp for the mutant allele. The different amplicon sizes for wild-type allele are due to presence or absence of a silent 4 bp polymorphic insertion to intronic sequences present in the amplicon.

### Whole-mount *in situ* hybridization

Single and double whole-mount *in situ* hybridizations were performed in glass vials as described (Paffett-Lugassy et al., 2013). Digoxygenin-labeled anti-sense riboprobes to *ltbp3* (Zhou et al., 2011), *ltbp1*, *nppa* and *thbs1a* were synthesized using a DIG RNA Labeling Kit (Roche Applied Science). The *ltbp1* probe template was generated by amplifying a cDNA sequence corresponding to exons 21-28 of the *ltbp1* locus. The amplicon was cloned into pCR4-TOPO to generate pCR4-TOPO*ltbp1*. For probe generation, pCR4-TOPO*ltbp1* was linearized with NotI and transcribed with T3 RNA polymerase. The *nppa* probe template was generated by amplifying a cDNA sequence containing a majority of the *nppa* coding sequence using the following primers: forward 5′-ACACGTTGAGCAGACACAGC-3′, reverse _T3 5′-aattaaccctcactaaaggTGTTAACAAATTAAGCCGTATTGT-3′ (lowercase letters indicate the promoter sequence of the T3 RNA polymerase). Because the reverse primer contained the T3 RNA promoter sequence (lower case), the amplicon was used directly in the DIG labeling reaction. The *thbs1a* probe template was generated by amplifying a cDNA sequence corresponding to exons 6-10 of the *thbs1a* locus. The amplicon was cloned into pCR4-TOPO to generate pCR4-TOPO*thbs1a.* For probe generation, pCR4-TOPO*thbs1a* was linearized with NotI and transcribed with T3 RNA polymerase. For single *in situ* hybridizations, a blue (NBT/BCIP) chromogenic substrate was utilized (Promega Corp.). Double *in situ* hybridizations were performed with a fluorescein-labeled antisense riboprobe to *cmlc2/myl7* (Yelon et al., 1999), which was synthesized using a Fluorescein RNA Labeling Kit (Roche Applied Science). A red (INT/ BCIP) chromogenic substrate was utilized (Roche Applied Science).

### Whole-mount immunostaining

Immunostaining was performed as described (Abrial et al. 2017). We used primary antibodies against the following proteins: GFP (B-2 mouse monoclonal antibody, catalog number sc-9996, Santa Cruz Biotechnology, 1:50 dilution); sarcomeric myosin heavy chain (MF20 mouse monoclonal antibody, Developmental Studies Hybridoma Bank, 1:50 dilution); tropoelastin 2 (anti-Eln2/Elnb rabbit polyclonal antibody, Miao et al., 2007, 1:1000 dilution); pSmad3 (rabbit monoclonal EP823Y against Smad3 phosphorylated at S423 and S425, catalog number ab52903, Abcam, 1:50 dilution) and pSmad1/5/9 (rabbit monoclonal antibody D5B10 against Smad1 phosphorylated at S463/465, Smad5 phosphorylated at S463/465 and Smad9 (Smad8) phosphorylated at S465/467; catalog number 13820, Cell Signaling Technologies; 1:100 dilution). Alexa-Fluor secondary antibodies (Alexa-Fluor-488 goat anti-mouse IgG, Alexa-Fluor-555 goat anti-rabbit IgG, Alexa-Fluor-647 goat anti-rabbit IgG; all Thermo Fisher Scientific) were used at 1:500 dilution. Nuclei were counterstained with DAPI using a 1:1000 dilution of a 1 mg/ml stock (Thermo Fisher Scientific).

### EdU incorporation

EdU (5-ethynyl-2′-deoxyuridine) labeling was performed using the Click-iT™ Plus EdU Cell Proliferation Kit (C10640; Thermo Fisher Scientific). Larvae were treated on 4-5 dpf with 1 mM EdU diluted in E3 medium and processed for whole-mount immunostaining as described (Abrial et al., 2017) with the following modifications: after fixation, bleaching and permeabilization, larvae were incubated in Click-iT^®^ Plus reaction cocktail for 1 h at RT in the dark. Larvae were washed several times with PBST prior to blocking and antibody staining as described (Abrial et al., 2017).

### Image analysis

Microscopic images were captured as described (Paffett-Lugassy et al., 2017). Unless otherwise stated, confocal images are maximum intensity *z*-stack projections. Cardiomyocytes were quantified in animals carrying the *cmlc2:nucGFP* transgene by manually labeling and tabulating GFP-positive nuclei while methodically scrolling through confocal *z*-stacks using Fiji software (Schindelin et al., 2012). Cardiomyocytes in the outflow tract were included in ventricular counts because distinguishing the two populations based on a molecular marker is not currently feasible. Ventricular areas, OFT diameters and OFT smooth muscle cell areas were measured in Fiji by first calibrating the measurement with the ‘set scale’ function and a known distance. To obtain the ventricular area, the ‘freehand selections’ tool was used to outline the perimeter of the ventricular wall in a maximum intensity *z*-stack projection. The area of the region within the perimeter was obtained using the ‘measure’ function. To obtain the OFT diameter, the largest diameter between the Eln2-positive smooth muscle walls, perpendicular to blood flow, was identified by scrolling through *z*-stacks. A line was drawn with the ‘straight’ tool and line length was obtained using the ‘measure’ function. Eln2-positive OFT smooth muscle cell sizes were measured as described for the ventricular area, except that cell perimeters were outlined in single optical sections of similar depths between experimental groups. Trabeculation was assessed in animals carrying the *cmlc2:nucGFP* transgene by comparing single optical sections of similar depths in the ventricular wall. The number of Eln2-positive OFT smooth muscle cells was quantified as described (Paffett-Lugassy et al., 2017). Eln2-positive cells containing EdU-positive nuclei were identified, and quantified manually by scrolling through confocal stacks of the OFT. The number of OFT endocardial cells was quantified in animals carrying the *fli1a:nEGFP* transgene by labeling and tabulating GFP-positive nuclei while methodically scrolling through confocal *z*-stacks using Fiji software. The proximal and distal boundaries of the OFT relative to the heart were determined by morphology. pSmad3 and pSmad1/5/9 intensities in the OFT were measured in confocal projections by first outlining the OFT with the ‘freehand selections’ tool and then obtaining the mean gray value from the ‘measure’ function. The average mean gray value from controls was used to calculate fold changes for the control (*n*=3/experiment) and mutant OFTs (*n*=3/experiment). The experiment was repeated three times. The signals in most confocal images shown were enhanced by adjusting the contrast and brightness in Fiji/ImageJ (Schindelin et al., 2012; Schneider et al., 2012). Exceptions to this are images showing pSmad3 and pSmad1/5/9 staining (Figs 4, 5, 7; Figs S7 and S9); these are raw, non-enhanced images because they were used to quantify signal intensity.

### Dissection of larval hearts and RNA extraction

Control and *ltbp1^−/−^; ltbp3^−/−^* larvae on 5 dpf were anesthetized in standard embryo medium containing 0.4% tricaine (ethyl 3-aminobenzoate methanesulfonate, MS222; Sigma) (Westerfield, 2000). Their hearts, including both the ventricle and OFT, were manually dissected using fine forceps and micro scissors (Fine Science Tools) and placed in cold 1×PBS (Westerfield, 2000). After collection of ten hearts per biological replicate, they were centrifuged at 4°C for 5 min at 16873 ***g*** before being resuspended in TRIzol Reagent (Thermo Fisher Scientific) and flash frozen in liquid nitrogen. Total RNA was isolated using the Direct-zol, RNA MicroPrep (Zymo Research, catalog number R2062) Kit, according to the manufacturer's instructions. RNA was eluted with 30 µl nuclease-free deionized water at room temperature.

### cDNA library preparation and RNA-sequencing analysis

RNA sample quality was evaluated using a 2100 Bioanalyzer Instrument (Agilent Technologies). Only samples scoring RIN>8 were used for cDNA library preparation. Approximately 400-500 ng of RNA per sample were used to prepare sequencing libraries with the low-input RNA NeoPrep Library Prep System (Illumina). Libraries were sequenced on a NextSeq500 system (Illumina; 40nt paired-end sequencing). For quality control purposes, reads were aligned against Zv9/danRer7 using Burrows-Wheeler Aligner (bwa-mem) version 0.7.12-r1039 (RRID:SCR_010910) with flags –t 16 –f and mapping rates, fraction of multiply-mapping reads, number of unique 20-mers at the 5′ end of the reads, insert size distributions and fraction of ribosomal RNAs were calculated using bedtools version 2.25.0.64 (RRID:SCR_006646) (Quinlan and Hall, 2010). In addition, each resulting bam file was randomly down-sampled to a million reads, which were aligned against Zv9/danRer7 and read density across genomic features were estimated for RNA-Seq-specific quality control metrics. Read mapping and quantification was performed using RSEM version 1.2.15 (RRID:SCR_013027), with rsem-calculate-expression command and flags -p 5 --output-genome-bam --paired-end --calc-ci --bowtie-chunkmbs 1024 against the Zv9/danRer7 genome assembly and ENSEMBL 70 annotation (bowtie version 1.0.1) (Langmead et al., 2009; Li and Dewey, 2011). Posterior mean estimates (PME) of counts and FPKM were retrieved for each sample. Differential expression analysis was performed using DESeq2 on count data from 4 control samples and 5 *ltbp1^−/−^; ltbp3^−/−^* samples, which produced log_2_-fold changes as well as raw and Benjamini–Hochberg adjusted *P*-values for each protein-coding gene (Love et al., 2014).

### Orthology analysis

ENSEMBL identities of probes on the Affymetrix Mouse GENE 2.1 array were retrieved from ENSEMBL 100 Biomart, resulting in 50,700 probes ID pairs, which corresponded to 33,396 unique murine IDs and 33,900 transcript clusters. Intersecting this set with the probes actually present in the Zilberberg et al. dataset (Zilberberg et al., 2015) (*n*=28,370), 37,611 probe-ID pairs remained (26,871 probe sets and 28,542 murine ENSEMBL IDs), of which 22,812 had a one-to-one relationship with an ENSEMBL ID, 1367 a one-to-many, 808 a many-to-one and 1884 a many to many. Probe IDs with a many-to-one or many-to-many relationship were re-assessed later in the analysis (including micro-RNAs, which were systematically discarded). Murine-Zebrafish orthology data were retrieved from ENSEMBL Biomart orthology server (ENSEMBL 100), yielding 21,599 ortholog pairs (17,347 unique Zebrafish IDS and 14,848 murine IDs). Intersecting them with the RNA-Seq data (*n*=25,999 IDs for protein-coding genes) yielded 17,024 ortholog pairs, corresponding to 13,842 zebrafish IDs and 12,631 murine ENSEMBL IDs. These ortholog pairs were subsequently intersected with the array data published by Zilberberg et al. (*n*=24,179, one-to-one and one-to-many Affy probe-ENSEMBL ID relationship) (Zilberberg et al., 2015), leading to 14,627 annotated ortholog pairs to be retained (13,065 zebrafish and 11,240 murine genes), and 11,418 murine ENSEMBL ID – Affymetrix probe pairs. Any ortholog pair/Affymetrix probe/ENSID with a relationship that was not one-to-one was set aside. Annotated zebrafish genes were scanned for genes with missing orthologs (thus presumably encompassing one-to-many and many-to-many genes) by intersecting the unassigned zebrafish ENSEMBL IDs against a Biomart query for murine, Zebrafish ENSEMBL IDs and Affymetrix MOUSE Gene 2.1 probes. This led to an additional 1565 ortholog pairs to be identified (1545 murine and 1460 Zebrafish IDs). Finally, all residual one-to-many and many-to-many pairs were manually curated based on the murine–zebrafish alignment scores, preservation of the gene order, ENSEMBL-based assignment of orthology, as well as matching of official gene symbols across species. When available, relevant comparative genomics literature was reviewed and gene families with multiple, many-to-many low-confidence assignments were discarded. Finally, redundant probe-level data were filtered to only retain unique exemplars of each, resulting in a final set of 11,269 ortholog pairs (9411 one-to-one, 1683 one-to-many and 175 many-to-many), corresponding to 10,570 unique murine ENSEMBL identities and 10,795 unique Zebrafish ENSEMBL identities.

### Pathway analysis

Zebrafish ENSEMBL identifiers for genes with adjusted *P*<0.05 and |fold changes| >1.5 were subject to pathway analysis using the DAVID webserver (Huang et al., 2009), with the set of expressed genes (baseMean value from DESeq2 above zero) used as a background. In the orthology analysis, DAVID was run on murine ENSIDs for genes with adjusted *P*<0.1 and |fold changes| >1.3× in both species, each quadrant separately, against a background of all ortholog pairs retained in the dataset (*n*=11,269). For Gene-set enrichment analysis (GSEA) (Mootha et al., 2003; Subramanian et al., 2005), Zebrafish-ENSEMBL ID were converted into Human ENSIDs based on an ENSEMBL Biomart orthology query (using ENSEMBL 100 version). For each human ENSID, matching Zebrafish fold changes were averaged and log2-transformed to generate a ranked expression list. This list was run against MsigDB version 7 sets c2, c3 and c5 with GSEA version 3.0 using parameters xtools.gsea.GseaPreranked -nperm 5000 -Xmx32 g -set_min 5 -set_max 2000 -plot_top_x 1000.

### Quantitative PCR analysis

First strand cDNA synthesis was achieved by using 1 µg of input RNA, purified from 10-20 5 dpf whole animals and using the SuperScript III First-Strand Synthesis System (Thermo Fisher Scientific). Quantitative real-time polymerase chain reaction (qRT-PCR) was performed in 96-well plates on a QuantStudio3 Real-Time PCR system (Thermo Fisher Scientific) using SYBR Green dye and gene-specific primers (Table S6). Four biological replicates and three technical replicates were analyzed. The 2^−ΔΔC_T_^ method (Livak and Schmittgen, 2001) was used to normalize expression to *rps11* and calculate relative expression levels between experimental groups.

### Small-molecule-mediated inhibition or activation of TGFβ signaling

The TGFβ type I receptor kinase antagonist LY364947 was sourced from Selleckchem or Sigma. A stock concentration of 10 mM was prepared in DMSO. Owing to minor batch-to-batch variation, the final working concentration was determined empirically for each batch, which was 10 µM or 20 µM based on the lowest concentration that would induce a robust phenotype. The TGFβ signaling agonist SRI-011381 was sourced from Selleckchem. A stock concentration of 100 mM was prepared in DMSO. The final working concentration was 500 µM, based on a dosing study to determine the highest non-lethal concentration of the drug. Healthy embryos without chorions were arrayed in clear multi-well plates on 2 dpf. Typically, 25 embryos were arrayed in 5 ml of E3 in 6-well plates or ten embryos were arrayed in 500 µl of E3 in 24-well plates. At the desired stage, a stock solution of LY364947, SRI-011381 or equivalent volumes of DMSO were added to each well to achieve the final working concentrations of LY364947 and SRI-011381. Plates were incubated at 28.5°C in a Ziploc bag containing a wet towel. For experiments taking longer than 24 h, fresh E3 and DMSO, LY364947 or SRI-011381 were added approximately every 24 h. Embryos were fixed and processed for immunostaining as described.

### Statistical analysis

Statistical analysis was performed with GraphPad Prism software version 7.00 for Macintosh. Differences between control and *ltbp1^−/−^; ltbp3^−/−^* animals were assessed by unpaired *t*-tests. All results are expressed as mean±one s.d. Investigators were blinded to cohort when quantifying cardiomyocyte, OFT smooth muscle or OFT endothelial cell numbers. All imaged embryos were included in the quantifications.

## Supplementary Material

Supplementary information
