# Supplementary material for: Latent TGFβ-binding proteins 1 and 3 protect the larval zebrafish outflow tract from aneurysmal dilatation
Source: Dis Model Mech. 2022 Mar 28;15(3):dmm046979. doi: 10.1242/dmm.046979 (PMC8990920; doi:10.1242/dmm.046979)
Supplement: Supplementary information [file dmm-15-046979-s1.pdf]

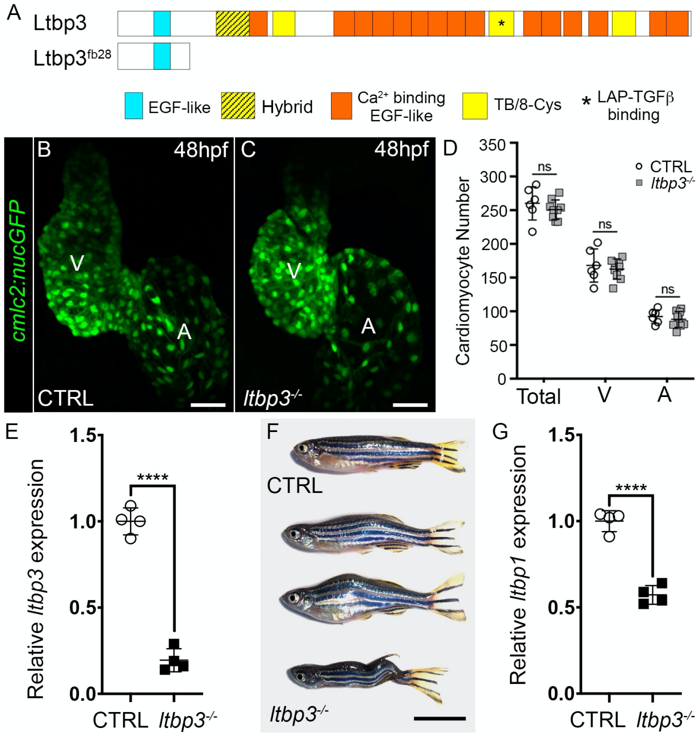

Fig. S1. Generation and characterization of *ltbp3* null zebrafish. (A) Schematic diagram showing the domain structure of zebrafish Ltbp3 (top; UniProtKB protein F1QFX6) and the predicted protein product encoded by the *ltbp3fb*^{28} allele (bottom), which contains 162 wild-type amino acids (of 1258 total) followed by 12 divergent amino acids (not shown) and a pre-mature termination codon. (B,C) Confocal projections of hearts from 48 hours post-fertilization (hpf) control (CTRL) and *ltbp3*$^{-/-}$ animals carrying the *Tg(cmlc2:nucGFP)* transgene immunostained with an antibody that recognizes GFP. Scale bars=30μm. (D) Dot plot showing the total, ventricular (V), and atrial (A) cardiomyocyte numbers in 48 hpf CTRL (n=6) and *ltbp3*$^{-/-}$ (n=9) embryos. Statistical significance was determined by unpaired t-tests and a Holm-Sidak's multiple comparisons test. (E) Dot plot showing the relative expression of *ltbp3* transcripts in CTRL and *ltbp3*$^{-/-}$ larvae on 5 days post-fertilization (dpf). n=4 biological replicates and 3 technical replicates. (F) Brightfield images of CTRL (top) and *ltbp3*$^{-/-}$ (bottom three) adult zebrafish. 10/10 null animals were affected by spinal curvatures. Scale bar=1cm. (G) Dot plot showing the relative expression levels of *ltbp1* transcripts in CTRL and *ltbp3*$^{-/-}$ larvae on 5 dpf. n=4 biological replicates and 3 technical replicates. In (E) and (G), *s*tatistical significance was determined by an unpaired t-test. For all dot plots, errors bars show one standard deviation. ns, not significant. ****, p<0.0001.

Disease Models & Mechanisms • Supplementary information

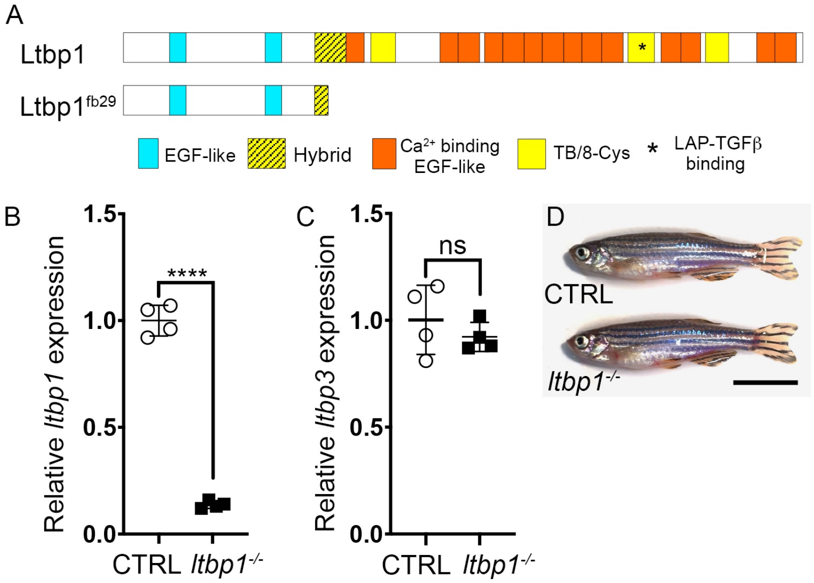

Fig. S2. Generation and characterization of *ltbp1* null zebrafish. (A) Schematic diagram showing the domain structure of zebrafish Ltbp1 (top; UniProtKB protein F1QQ56) and the predicted protein product encoded by the *ltbp1fb*^{29} allele (bottom), which contains 426 wild-type amino acids (of 1428 total) followed by 16 divergent amino acids (not shown) and a pre-mature termination codon. (B,C) Dot plots showing the relative expression levels of *ltbp1* and *ltbp3* transcripts in control (CTRL) and *ltbp1*$^{-/-}$ larvae on 5 days post-fertilization (dpf). Statistical significance was determined by an unpaired t-test. Errors bars show one standard deviation. ****, p<0.0001. ns, not significant. n=4 biological replicates and 3 technical replicates. (D) Brightfield images of adult CTRL (top) and *ltbp1*$^{-/-}$ (bottom) zebrafish. Little to no variation was observed between animals within each group (n>10/group). Scale bar=1cm.

Disease Models & Mechanisms • Supplementary information

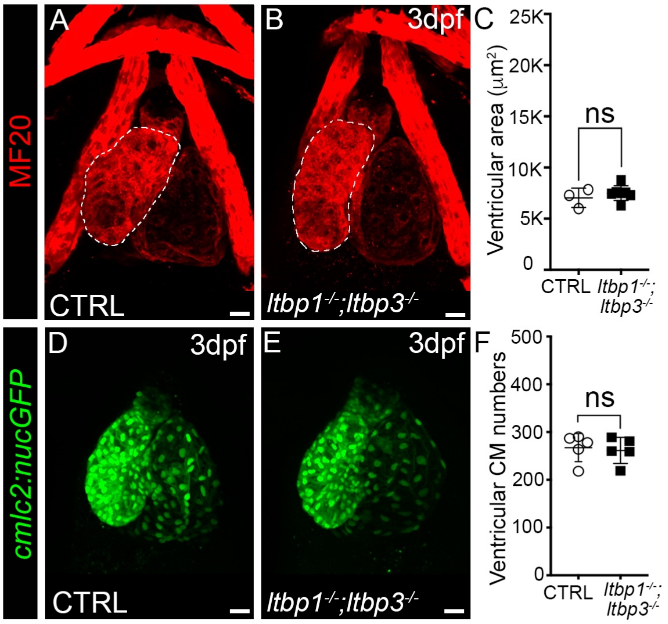

Fig. S3. Ventricular morphogenesis is unperturbed in *ltbp1, ltbp3* double knockout embryos. (A,B) Confocal projections of hearts in 3 days post-fertilization (dpf) control (CTRL; A) and *ltbp1$^{-/-}$; ltbp3$^{-/-}$* (B) larvae double immunostained with an antibody (MF20) that recognizes striated muscle. Ventricular size was measured by quantifying the area enclosed by the chamber's perimeter [shown as dotted lines in (A,B)]. (C) Dot plot showing the ventricular areas of CTRL (n=3) and *ltbp1$^{-/-}$; ltbp3$^{-/-}$* (n=7) larvae. (D,E) Confocal projections of hearts in 3 dpf CTRL (D) and *ltbp1$^{-/-}$; ltbp3$^{-/-}$* (E) larvae carrying the *cmlc2:nucGFP* transgene immunostained with an antibody that recognizes GFP. (F) Dot plot showing the numbers of ventricular cardiomyocytes in 3 dpf CTRL (n=5) and *ltbp1$^{-/-}$; ltbp3$^{-/-}$* (n=5) larvae. For (C) and (F), statistical significance was determined by an unpaired t-test. Error bars show one standard deviation. ns, not significant. Scale bars=20μm.

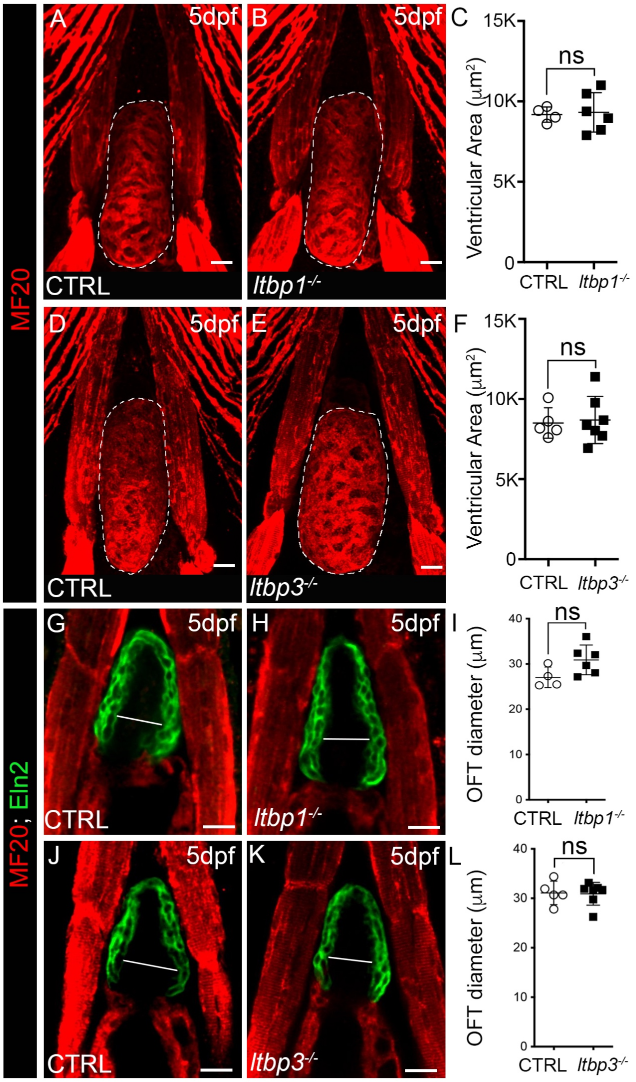

Fig. S4. Absence of OFT aneurysm and ventricular dilation in 5 dpf *ltbp1* null and *ltbp3* null single-mutant zebrafish. (A,B,D,E) Confocal projections of hearts in 5 days post-fertilization (dpf) control (CTRL; A,D), *ltbp1*$^{-/-}$ (B), and *ltbp3*$^{-/-}$ (E) larvae immunostained with an antibody (MF20) that recognizes striated muscle. Ventricular size was measured by quantifying the area enclosed by the chamber's perimeter [shown as dotted lines in (A,B,D,E)]. (C,F) Dot plots showing the ventricular areas in CTRL [n=4 in (C); n=5 in (F)], *ltbp1*$^{-/-}$ (C, n=6), and *ltbp3*$^{-/-}$ (F, n=7) larvae. (G,H,J,K) Single optical sections through the OFTs of 5 dpf CTRL (G,J), *ltbp1*$^{-/-}$ (H), and *ltbp3*$^{-/-}$ (K) larvae double immunostained with antibodies that recognize striated muscle (MF20, red) or Eln2+ OFT smooth muscle (green). The white lines show the maximal OFT diameters between the Eln2 + smooth muscle that are perpendicular to blood flow. (I,L) Dot plots showing the maximal OFT diameters in 5 dpf CTRL [n=4 in (I); n=5 in (L)], *ltbp1*$^{-/-}$ [n=6 in (I)], and *ltbp3*$^{-/-}$ [n=6 in (L)] larvae. For all dot plots, statistical significance was determined by an unpaired t-test. Error bars show one standard deviation. ns, not significant. Scale bars=20μm.

Disease Models & Mechanisms • Supplementary information

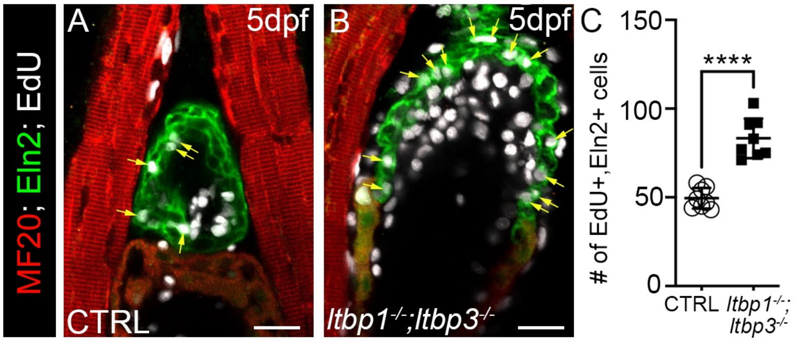

Fig. S5. OFT smooth muscle cell hyperplasia in *Itbp1*, *Itbp3* double knockout animals. (A,B) Single optical sections through the OFTs of 5 days post-fertilization (dpf) control (CTRL; A) and *Itbp1*$^{-/-}$; *Itbp3*$^{-/-}$ (B) larvae exposed to EdU between 4 and 5 dpf, processed for Click-iT EdU labelling (white), and double immunostained with antibodies that recognize striated muscle (MF20, red) or Eln2+ OFT smooth muscle (green). (C) Dot plot showing the numbers of EdU+, Eln2+ cells in CTRL (n=8) and *Itbp1*$^{-/-}$; *Itbp3*$^{-/-}$ OFTs (n=8). Statistical significance was determined with an unpaired t-test. Error bars show one standard deviation. ****, p<0.0001. Scale bars=20μm.

Disease Models & Mechanisms • Supplementary information

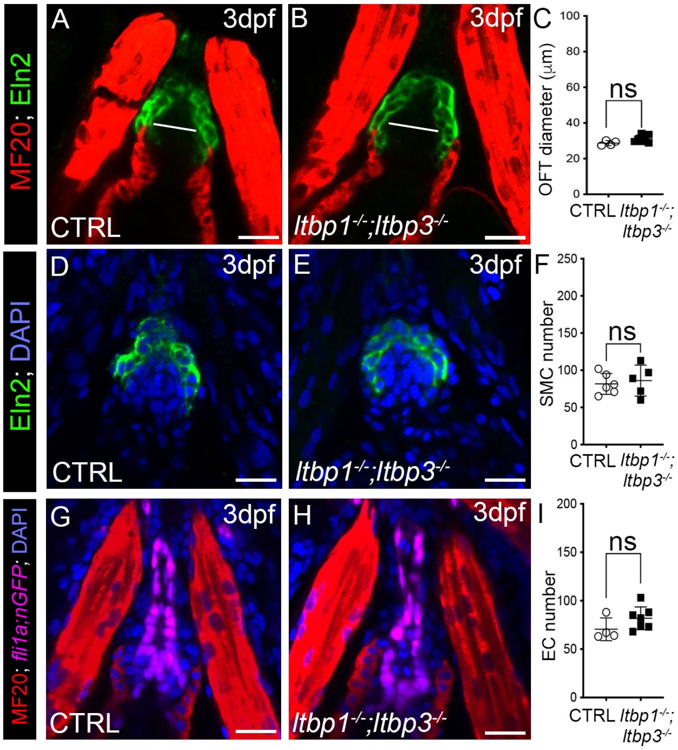

Fig. S6. OFT morphogenesis is unperturbed in *ltbp1, ltbp3* double knockout embryos. (A,B) Single optical sections through the OFTs of 3 days post-fertilization (dpf) control (CTRL; A,D) and *ltbp1$^{-/-}$; ltbp3$^{-/-}$* (B,E) larvae double immunostained with antibodies that recognize striated muscle (A,B; MF20, red) or Eln2+ OFT smooth muscle (A,B,D,E; green) and counterstained with DAPI (D,E; blue). (C,F) Dot plots showing the maximal OFT diameters and Eln2+ OFT smooth muscle cell (SMC) numbers in 3 dpf CTRL [n=4 in (C); n=6 in (F)] and *ltbp1$^{-/-}$; ltbp3$^{-/-}$* [n=7 in (C); n=5 in (E)] larvae. The white lines in (A,B) show the maximal OFT diameters between the Eln2+ smooth muscle that are perpendicular to blood flow. SMC number was quantified by counting the number of DAPI stained nuclei surrounded by Eln2+ signal. (G,H) Single optical sections of OFTs from 3 dpf CTRL and *ltbp1$^{-/-}$; ltbp3$^{-/-}$* larvae carrying the endothelial/endocardial *fli1a:nGFP* transgene immunostained with antibodies that recognize striated muscle (MF20, red) or GFP (magenta) and counterstained with DAPI (blue). (I) Dot plot showing the numbers of endocardial cells in the OFTs of 3 dpf CTRL (n=4) and *ltbp1$^{-/-}$; ltbp3$^{-/-}$* (n=7) larvae. For all dot plots, statistical significance was determined with an unpaired t-test. Error bars show one standard deviation. ns, not significant. Scale bars=20μm.

Disease Models & Mechanisms • Supplementary information

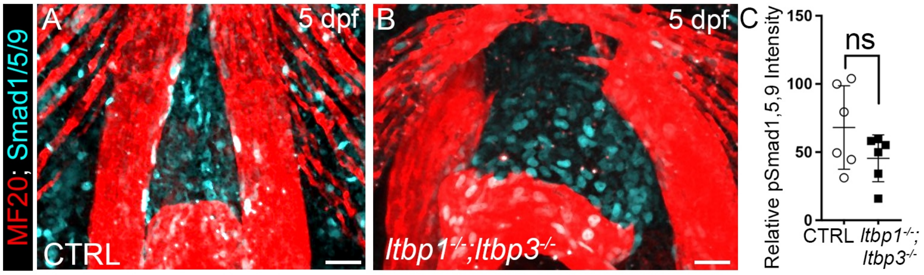

Fig. S7. The distended OFTs of *ltbp1*, *ltbp3* double knockout animals do not hyperactivate BMP signaling. (A,B) Confocal projections of OFTs in 5 days post-fertilization (dpf) control (CTRL; A) and *ltbp1$^{-/-}$; ltbp3$^{-/-}$* (B) larvae double immunostained with antibodies that recognize striated muscle (MF20, red) or pSmad1/5/9 (cyan). Dot plot showing the relative mean pSmad1/5/9 fluorescence intensities in the OFTs of 5 dpf CTRL (n=6) and *ltbp1$^{-/-}$; ltbp3$^{-/-}$* (n=6) larvae. Statistical significance was determined with an unpaired t-test. Error bars show one standard deviation. ns, not significant. Scale bars=20μm.

Disease Models & Mechanisms • Supplementary information

A

	Collection	Functional Category	Fold Enrichment	BH-adj p-value	Example genes
Upregulated	GOTERM_BP	protein folding	4.83	2.97E-04	*ranbp2, fkbp14, calrl2, fkbp7, bag2, fkbp9, pdip5, dnajb11, nudcd2, dnajb1b, hsp90b1, hspd1, dnaja1l, pdia4, dnajb5, fkbp1ab*
	KEGG_PATHWAY	Ribosome biogenesis in eukaryotes	4.97	1.89E-03	*nol6, gar1, gnl3, imp4, xpo1a, imp3, smfn, fbl, pwp2h, bms1l, nat10*
	SMART	SERPIN	8.22	8.45E-03	*serpine3, serpina7, serpina1l, serpinh1b, serpine1*
	KEGG_PATHWAY	AA-tRNA biosynthesis	5.85	0.015	*larsb, nars, vars, qars, yars, tars, pars2*
	UP_KEYWORDS	Mitochondrion	2.34	0.038	*gfm1, tsfm, pif1, bcs1l, slc25a25b, timm13, cycsb, gfm2, mpv17l2, samm50, slc25a38b, alas2, agk, chchd4, apex1, timm8b, supv3l1, qtrtd1, sco2, oma1*
	GOTERM_BP	rRNA processing	4.91	0.046	*nol6, gar1, nop2, imp4, exosc6, imp3, fbl, bms1l, nat10*
	UP_KEYWORDS	DNA damage	3.79	0.051	*msh3, pif1, prmt6, apex1, ube2t, tipin, nabp1a, ruvbl2, smarcad1*
	GOTERM_MF	oxygen binding	8.96	0.054	*hbm, hbae1, hbz, hbbe2, hbae1*
	GOTERM_MF	unfolded protein binding	4.65	0.059	*nudcd2, dnajb11, trap1, dnajb1b, hsp90b1, dnaja1l, calrl2, dnajb5*
Downregulated	KEGG_PATHWAY	Metabolic pathways	2.02	9.22E-12	*ivd, gamt, tpi1b, agmat, acsl4a, pah, cecr1a, ahcy, oat, pgam1a, hgd, cbsa, got2a, aldob*
	GOTERM_MF	oxidoreductase activity	2.60	3.58E-07	*dhdh, hgd, cyp2n13, alkbh2, cyp27a7, aoc1, aldh9a1a.2, cyp4t8, CYP46A1, hpda, mdh1a, dio1, cyp4v7*
	KEGG_PATHWAY	Biosynthesis of amino acids	5.88	4.32E-07	*tpi1b, pah, pgam1a, cbsa, idh1, got2a, pc, aldob, shmt1, tat, SDSL, acy1, cbsb, got1, gapdh, bcat1*
	GOTERM_BP	gluconeogenesis	11.30	7.18E-04	*tpi1b, pgam1a, pck1, g6pca.2, pc, g6pca.1, fbp1b*
	GOTERM_MF	transaminase activity	9.62	4.54E-03	*oat, agxta, got1, bcat1, got2a, agxtb*
	GOTERM_MF	metalloaminopeptidase activity	8.61	8.08E-03	*anpepa, enpep, xpnpep2, lta4h, metap1, xpnpep1*
	GOTERM_CC	troponin complex	7.98	0.012	*tnni2a.4, tnni2b.2, tnni1al, zgc:153662, tnnt1, zgc:92233*
	KEGG_PATHWAY	PPAR signaling pathway	3.86	0.014	*acsl4a, cd36, aqp7, acsl5, pck1, scp2a, acox3, cyp7a1a*
	KEGG_PATHWAY	Adipocytokine signaling pathway	3.31	0.019	*acsl4a, IRS2, cd36, acsl5, nfkbie, pck1, g6pca.2, nfkbiab, g6pca.1*
	GOTERM_MF	neutral amino acid transmembrane transporter activity	10.62	0.045	*slc15a1b, slc43a2b, CU571169.1, CU856173.1*
	GOTERM_BP	cellular response to estrogen stimulus	6.72	0.049	*ucp1, serp1, nupr1, pah, agxtb, fbp1b*

B

Collection	Functional Category	Fold Enrichment	BH-adj p-value	Example Genes
KEGG_PATHWAY	Complement and coagulation cascades	18.06	1.08E-06	*C2, C5ar1, F2r, C3ar1, Plat, C4b, C1qb, Serpine1, C1qc*
UP_KEYWORDS	Immunity	8.70	3.97E-06	*C2, Tlr3, Cd84, Myo1g, Tlr8, Havcr2, C4b, C1qb, Syk, Slamf7, H2-Eb1, Tmem173, C1qc*
GOTERM_BP_DIRECT	inflammatory response	8.75	7.91E-05	*Camk1d, Tlr3, C5ar1, F2r, C3ar1, Cybb, Agtr1a, Tlr8, Havcr2, C4b, Adam8, Alox5*
UP_KEYWORDS	Cell adhesion	5.66	4.04E-04	*Siglece, Emilin2, Cd84, Itgax, Myh9, Fermt3, Fn1, Cd33, Vmp1, Itgb2, Cd44, Itga6*
UP_KEYWORDS	Glycoprotein	1.97	1.79E-03	*Cd53, C5ar1, Angpt2, Piezo2, Gria4, Ptprc, Serpine1, H6pd, Siglece, Emilin2, Lyve1, Cd84, Itgax, Fn1, Cd33, Cd44, Itga6, C2, C3ar1, Has1, Plat, C1qb, Itgb2, H2-Eb1, Tlr3, Mpeg1, F2r, Cybb, Agtr1a, Tlr8, Havcr2, C4b, Adam8, Slamf7*
UP_SEQ_FEATURE	signal peptide	2.32	2.01E-03	*Angpt2, Gria4, Ptprc, Serpine1, H6pd, Siglece, Emilin2, Lyve1, Cd84, Itgax, Fn1, Cd33, Mdk, Cd44, Itga6, C2, Plat, C1qb, Itgb2, H2-Eb1, C1qc, Tlr3, Mpeg1, F2r, Tlr8, Havcr2, Tmed5, C4b, Adam8, Slamf7*
UP_KEYWORDS	Cell division	4.35	6.56E-03	*Cdk6, Aurka, Mcm5, Ccnb1, Sept3, Fam83d, Cdk1, Ccna2, Tacc3, Mad2l1*
UP_KEYWORDS	Calmodulin-binding	8.17	9.85E-03	*Camk1d, Myh9, Myo1g, Cnn2, Myo1f, Marcks*
UP_KEYWORDS	Cell cycle	3.08	0.018	*Cdk6, Aurka, Mcm5, Mki67, Ccnb1, Sept3, Fam83d, Cdk1, Ccna2, Tacc3, Mad2l1, Ikzf1*
GOTERM_CC_DIRECT	integrin complex	23.90	0.021	*Itgax, Myh9, Itgb2, Itga6*
GOTERM_CC_DIRECT	extracellular exosome	2.04	0.022	*Fabp5, Cd53, Fermt3, Cdk1, Ptprc, Rnd3, Serpine1, Lyve1, Nudt14, Cd84, Myo1g, Fn1, Cnn2, Cd44, Marcks, Twf2, C2, Myh9, Plat, C1qb, Itgb2, Coro1a, H2-Eb1, C1qc, Psat1, Rab8b, Sphkap, Havcr2, Fmnl1, C4b*
UP_KEYWORDS	Actin-binding	4.98	0.025	*Twf2, Myh9, Myo1g, Cnn2, Myo1f, Coro1a, Marcks*
GOTERM_BP_DIRECT	organ regeneration	17.05	0.026	*Angpt2, C5ar1, Mki67, Cdk1, Ccna2*
KEGG_PATHWAY	Hematopoietic cell lineage	13.61	0.047	*Cd33, Cd44, H2-Eb1, Itga6*
KEGG_PATHWAY	Osteoclast differentiation	6.61	0.048	*Spi1, Ncf2, Cybb, Mapk11, Junb, Syk*
GOTERM_BP_DIRECT	positive regulation of angiogenesis	8.69	0.049	*Angpt2, C5ar1, C3ar1, Cybb, Itgb2, Serpine1*

Fig. S8. Gene Ontology term enrichment analysis of RNA-sequencing data from *Itbp1*, *Itbp3* double knockout disease tissue. (A) Table showing representative Gene Ontology (GO) terms enriched in the upregulated (red dots in Fig. 6A) or downregulated (blue dots in Fig. 6A) gene sets in *Itbp1*$^{-/-}$; *Itbp3*$^{-/-}$ OFTs and ventricles. Inclusion criteria were |fold change (FC)|>1.5 and adjusted p-value<0.05. (B) Table showing representative GO terms enriched in orthologous gene pairs upregulated in disease tissue from *Fbn1*$^{mgR/mgR}$ mice and *Itbp1*$^{-/-}$; *Itbp3*$^{-/-}$ zebrafish (red dots in upper right quadrant of Fig. 6G) using the mouse gene identifiers as inputs. Inclusion criteria were |FC|>1.3 and adjusted p-value<0.1 for both orthologs within each pair.

Disease Models & Mechanisms • Supplementary information

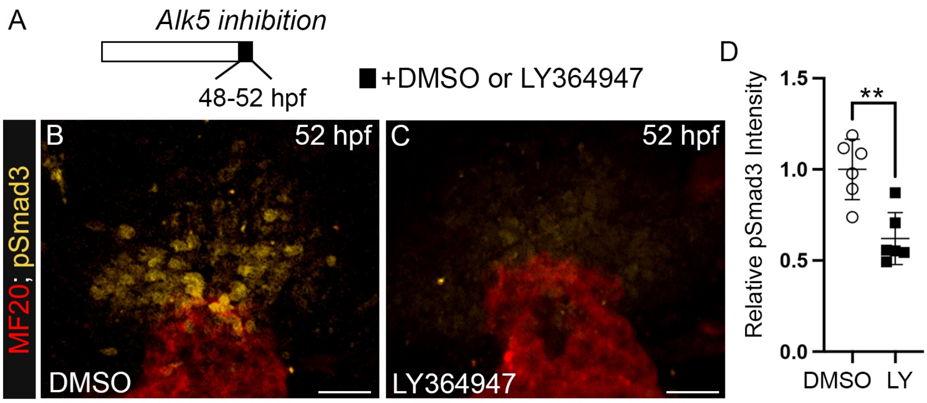

Fig. S9. Validation of LY364947 as an antagonist of TGFβ signaling in the zebrafish OFT. (A) Experimental timeline for small molecule-mediated inhibition of TGFβ signaling in wild-type animals. (B,C) Confocal projections of OFTs in 52 hours post-fertilization (hpf) wild-type animals treated with DMSO (B) or LY364947 (C) and doubled immunostained with antibodies that recognize striated muscle (MF20, red) or phosphorylated Smad3 (pSmad3; green). (D) Dot plot showing the relative mean pSmad3 fluorescence intensities in the OFTs of 52 hpf wild-type embryos treated with DMSO (n=6) or LY364947 (n=6). Statistical significance was determined with an unpaired t-test. Error bars indicate one standard deviation. **, $p<0.01$. Scale bars=20μm.

Disease Models & Mechanisms • Supplementary information

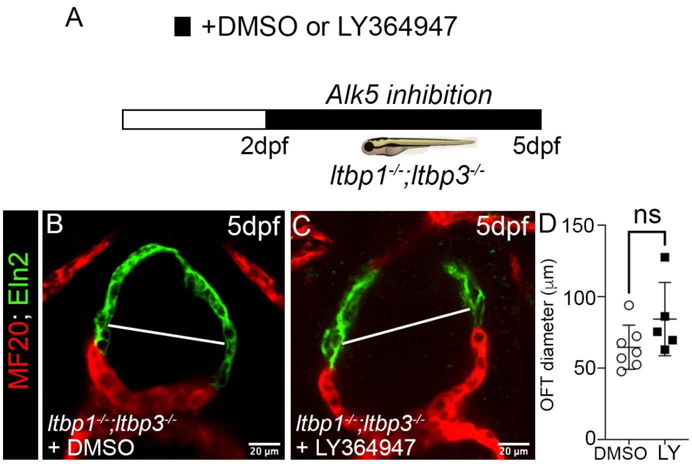

Fig. S10. Inhibition of TGFβ signaling does not suppress aneurysm in *Itbp1$^{-/-}$*; *Itbp3$^{-/-}$* animals. (A) Experimental timeline for small molecule-mediated inhibition of TGFβ signaling in *Itbp1$^{-/-}$*; *Itbp3$^{-/-}$* animals. (B,C) Single optical sections of OFTs in 5 days post-fertilization (dpf) wild-type animals treated with DMSO (B) or LY364947 (C) and double immunostained with antibodies that recognize striated muscle (MF20, red) or Eln2+ OFT smooth muscle (green). (D) Dot plot showing the maximal OFT diameters of 5 dpf *Itbp1$^{-/-}$*; *Itbp3$^{-/-}$* larvae treated with DMSO (n=7) or LY364947 (n=5). Statistical significance was determined with an unpaired t-test. Error bars indicate one standard deviation. **, p<0.01. Scale bars=20μm.

Disease Models & Mechanisms • Supplementary information

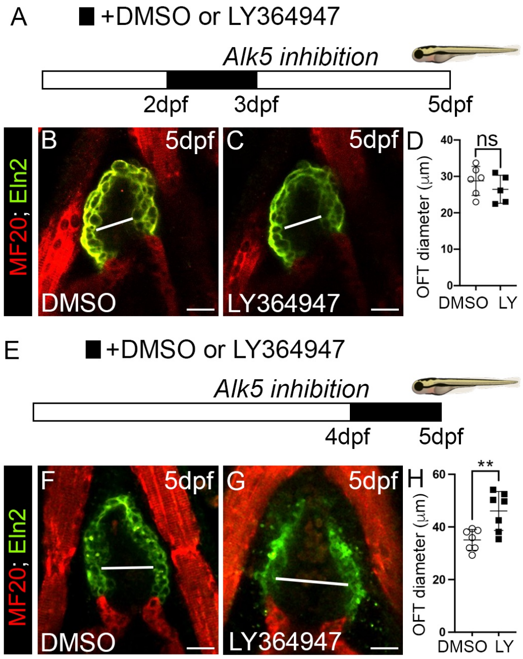

Fig. S11. TGFβ signaling protects the zebrafish OFT from aneurysm subsequent to co-expression of *ltbp3* and *ltbp1*. (A) Experimental timeline for small molecule-mediated inhibition of TGFβ signaling. (B,C) Single optical sections of OFTs in 5 dpf wild-type animals treated with DMSO (B) or LY364947 (C) as shown in (A) and double immunostained with antibodies that recognize striated muscle (MF20, red) or Eln2+ OFT smooth muscle (green). (D) Dot plot showing the maximal OFT diameters of 5 dpf wild-type larvae treated with DMSO (n=6) or LY364947 (n=5) on 2-3 dpf. (E) Experimental timeline for small molecule-mediated inhibition of TGFβ signaling in wild-type animals. Single optical sections of OFTs in 5 dpf wild-type animals treated with DMSO (F) or LY364947 (G) as shown in (E) and double immunostained with antibodies that recognize striated muscle (MF20, red) or Eln2+ OFT smooth muscle (green). (H) Dot plot showing the maximal OFT diameters of 5 dpf wild-type larvae treated with DMSO (n=7) or LY364947 (n=7) on 4-5 dpf. For all dot plots, statistical significance was determined with an unpaired t-test. Error bars indicate one standard deviation. ns, not significant. **, $p<0.01$. Scale bars=20μm

Disease Models & Mechanisms • Supplementary information

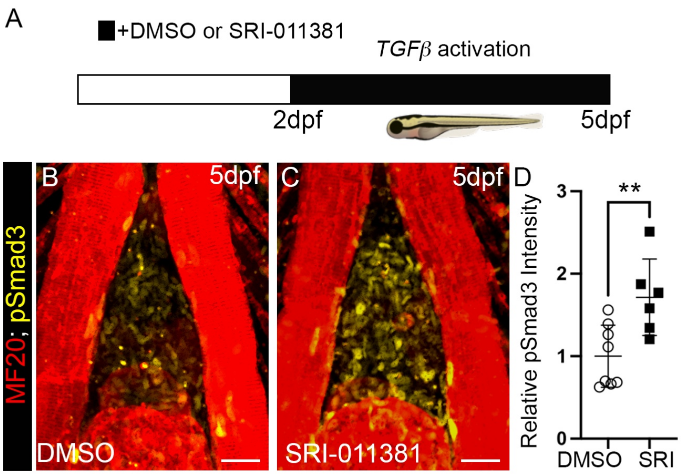

Fig. S12. Validation of SRI-011381 as an agonist of TGFβ signaling in the zebrafish OFT. (A) Experimental timeline for small molecule-mediated activation of TGFβ signaling in wild-type animals. (B,C) Confocal projections of OFTs in 5 days post-fertilization (dpf) wild-type animals treated with DMSO (B) or SRI-011381 (C) and immunostained with antibodies that recognize striated muscle (MF20, red) or phosphorylated Smad3 (pSmad3; green). (D) Dot plot showing the relative mean pSmad3 fluorescence intensities in the OFTs of 5 dpf wild-type embryos treated with DMSO (n=8) or SRI-011381 (n=6). Statistical significance was determined with an unpaired t-test. Error bars indicate one standard deviation. **, $p<0.01$. Scale bars=20μm.

Disease Models & Mechanisms • Supplementary information

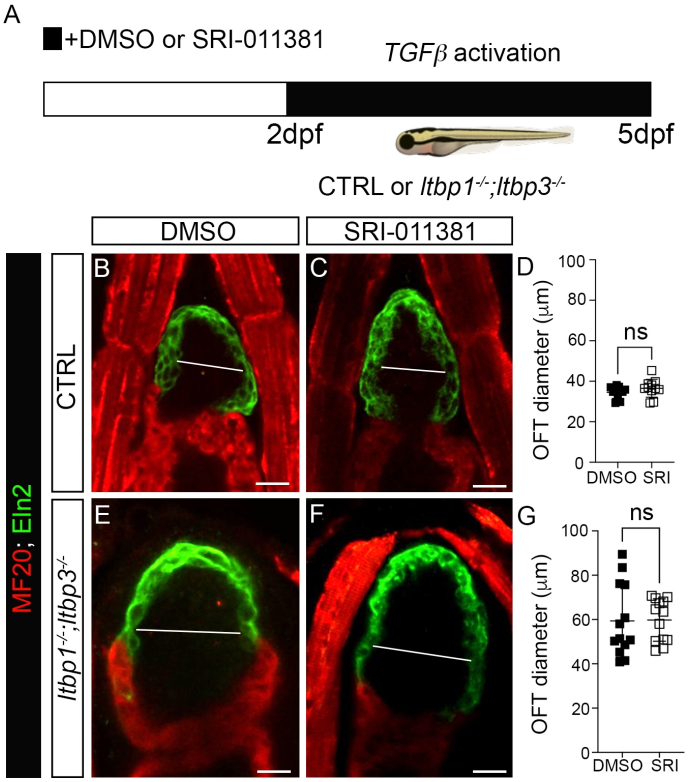

Fig. S13. Activation of TGFβ signaling does not induce OFT aneurysm in control-sibling animals or suppress OFT aneurysm in *Itbp1$^{-/-}$*; *Itbp3$^{-/-}$* animals. (A) Experimental timeline for small molecule-mediated activation of TGFβ signaling in control-sibling (CTRL) and *Itbp1$^{-/-}$*; *Itbp3$^{-/-}$* animals. (B,C,E,F) Single optical sections of OFTs in 5 days post-fertilization (dpf) CTRL (B,C) and *Itbp1$^{-/-}$*; *Itbp3$^{-/-}$* (E,F) larvae treated with DMSO (B,E) or SRI-011381 (C,F) and double immunostained with antibodies that recognize striated muscle (MF20, red) or Eln2+ OFT smooth muscle (green). (D,G) Dot plots showing the maximal OFT diameters of 5 dpf CTRL (D) and *Itbp1$^{-/-}$*; *Itbp3$^{-/-}$* (G) larvae treated with DMSO [n=12 in (D); n=13 in (G)] or SRI-011381 [n=11 in (D); n=13 in (F)]. Statistical significance was determined with an unpaired t-test. Error bars indicate one standard deviation. ns, not significant. Scale bars=20μm.

Disease Models & Mechanisms • Supplementary information

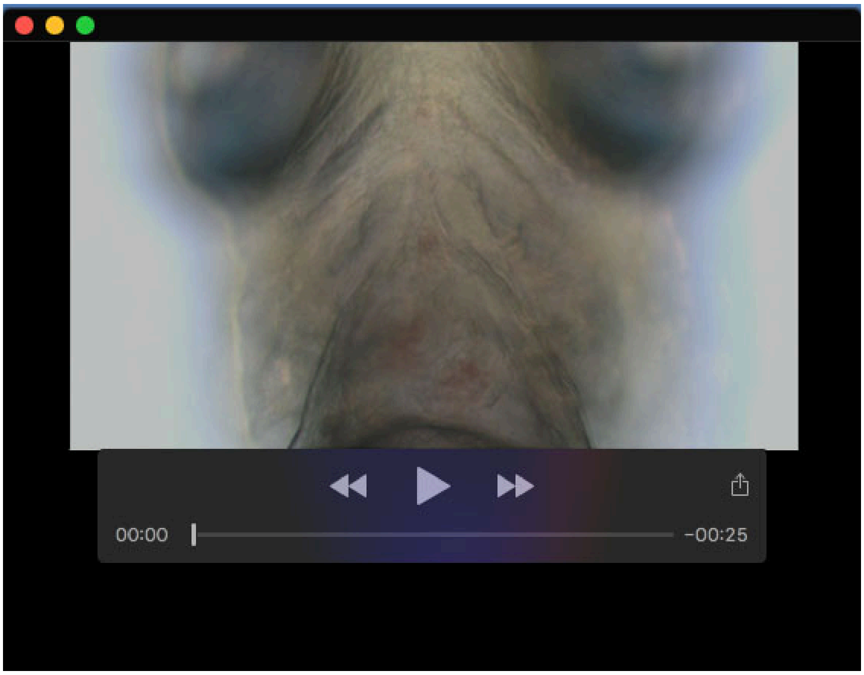

Movie 1. Brightfield video of 5 days post-fertilization control-sibling heart**.**

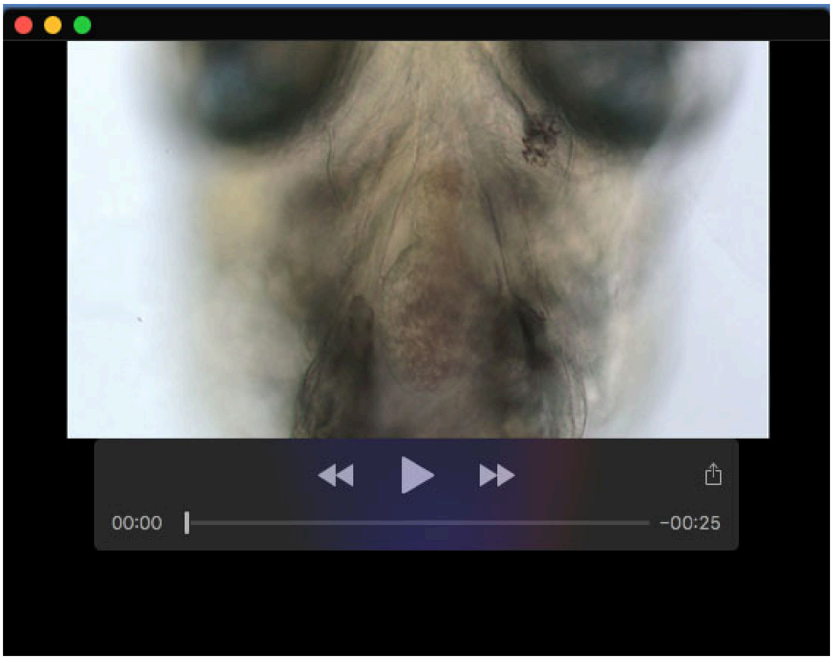

Movie 2. Brightfield video of 5 days post-fertilization *ltbp1*$^{-/-}$; *ltbp3*$^{-/-}$ heart showing aortic regurgitation.

Table S1. Genes differentially expressed between co-dissected ventricles and OFTs from *Itbp1*$^{-/-}$; *Itbp3*$^{-/-}$ and control animals.

Click here to download Table S1

Table S2. Gene ontology (GO) terms for genes differentially expressed between co-dissected ventricles and OFTs from *Itbp1-/-; Itbp3-/-* and control animals, |FC|>1.5, adjusted p-value<0.05.

Click here to download Table S2

Table S3. Gene set enrichment analysis (GSEA) of human homologs of genes differentially expressed between co-dissected ventricles and OFTs from *Itbp1-/-; Itbp3-/-* and control animals, MsigDB v. 7.0, c2, c3 and c5.

Click here to download Table S3

Table S4. Orthology analysis of genes differentially expressed in disease tissue from *Fbn1*$^{mgR/mgR}$ mice and *Itbp1*$^{-/-}$; *Itbp3*$^{-/-}$ zebrafish, |FC|>1.3, adjusted p-value<0.1.

Click here to download Table S4

Table S5. Gene ontology (GO) terms for orthologous gene pairs behaving similarly in disease tissue from *Fbn1*$^{mgR/mgR}$ mice and *Itbp1*$^{-/-}$; *Itbp3*$^{-/-}$ zebrafish, |FC|>1.3, adjusted p-value<0.1.

Click here to download Table S5

Disease Models & Mechanisms • Supplementary information

Table S6. qPCR Primers.

qPCR primers	Forward primer (5′ to 3′)	Reverse Primer (5′ to 3′)
ltbp3	ctggttatcagcctacgcaag	ggcagctcgtttacactcgt
ltbp1	cctgatttctggagatcctatgag	gctggcattcatctgcat
nppa	caacatggccaagctcaa	ggctctctctgatgcctcttc
nppb	aacgacgacatgggtgtttt	tttgccgcctttacttctct
tgfb1b	caagtgggtgtcgtttgatg	cagctgaaactcctgcttctc
tgbr2a	cgacagctgcctgagtcc	catataacgcgccgttcc
thbs1a	acgcaagagtgtgacaaacg	gaggaccagggagaccag
serpine1	gagcagaatgggtcttggag	ttggatacacataaaggttcctca
rps11	gatggcggacactcagaac	ccaatccaacgtttctgtga
